# Solid Lipid Nanoparticles Coated with Glucosylated poly(2-oxazoline)s: A Supramolecular Toolbox Approach

**DOI:** 10.1021/acs.biomac.4c01052

**Published:** 2025-01-08

**Authors:** Johanna K. Elter, František Sedlák, Tomáš Palušák, Nicol Bernardová, Volodymyr Lobaz, Eva Tihlaříková, Vilém Neděla, Pavel Šácha, Martin Hrubý

**Affiliations:** † Institute of Macromolecular Chemistry, CAS, Heyrovského nám. 2, Praha 6 162 06, Czech Republic; ‡ Institute of Biochemistry and Experimental Oncology, First Faculty of Medicine, U Nemocnice 5, Praha 2 128 53, Czech Republic; § Institute of Scientific Instruments, CAS, Královopolská 147, Brno 612 00, Czech Republic; ∥ Institute of Organic Chemistry and Biochemistry, CAS, Flemingovo nám. 2, Praha 6 166 10, Czech Republic

## Abstract

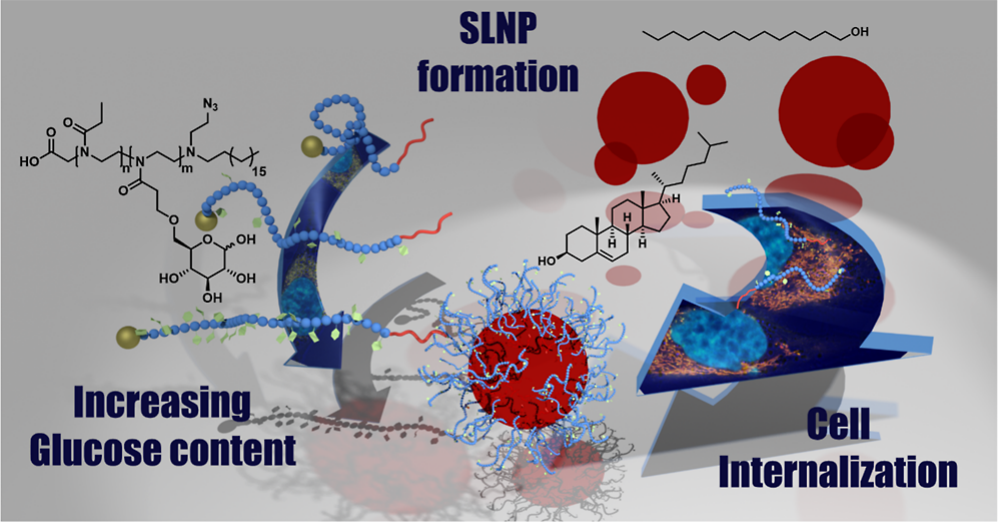

Multifunctional polymers are interesting substances for the formulation of drug molecules that cannot be administered in their pure form due to their pharmacokinetic profiles or side effects. Polymer-drug formulations can enhance pharmacological properties or create tissue specificity by encapsulating the drug into nanocontainers, or stabilizing nanoparticles for drug transport. We present the synthesis of multifunctional poly(2-ethyl-2-oxazoline-*co*-2-glyco-2-oxazoline)s containing two reactive end groups, and an additional hydrophobic anchor at one end of the molecule. These polymers were successfully used to stabilize (solid) lipid nanoparticles ((S)LNP) consisting of tetradecan-1-ol and cholesterol with their hydrophobic anchor. While the pure polymers interacted with GLUT1-expressing cell lines mainly based on their physicochemical properties, especially *via* interactions of the hydrophobic anchor with membranous compartments of the cells, LNP-cell interactions hinted toward an influence of the glucosylation on particle–cell interactions. The presented LNP are therefore promising systems for the delivery of drugs into GLUT1-expressing cell lines.

## 1 Introduction

Latest with the COVID-19 pandemic and the development of RNA vaccines, it became apparent that the formulation of a physiologically active compound can be at least as important as the compound itself.^1−4^ During the last decades, the generation of suitable drug formulations for specific applications was the focus of numerous chemists. Depending on the nature of the active molecule, protection of the latter against degradation in physiological environments or recognition by the immune system may be necessary.^4,5^ Further, drug molecules can exhibit unwanted toxicity or side effects during circulation in the bloodstream if they distribute randomly throughout different tissues in the body or accumulate in an undesired location (e.g, liver).^2,4,6^ These obstacles can be overcome by encapsulating drug molecules within nanostructures,^7^ or even covalently attach them to the latter.^8^ The choice of a suitable nanostructure depends on different chemical properties of the drug molecule and its target.

For example, drug molecules that are hydrophobic and therefore exhibit insufficient solubility under physiological conditions, can be encapsulated into polymeric micelles or lipid nanoparticles (LNP).^4,9^ Both structures consist of a hydrophobic core that can incorporate hydrophobic compounds, and a hydrophilic shell that solubilizes the core (and the drug molecule) in aqueous media. Polymeric micelles can be generated from amphiphilic block copolymers and exhibit a large versatility when it comes to the choice of building blocks–the monomers for the hydrophobic block, for example, can be chosen to maximize drug loading efficiency,^10^ enable covalent attachment of the drug,^11^ or influence micellar stability.^12^ The choice of suitable hydrophilic monomers ensures sufficient solubilization of the structure as well as low toxicity and recognition by the immune system.^13^ However, the long-term fate of many polymers commonly used as building blocks for polymeric micelles in drug delivery remains unclear. Not all of them are biodegradable, therefore accumulation of polymeric micelles or polymer fragments within the body may occur.^14,15^

LNP on the other hand are nanosized lipid droplets that are stabilized by amphiphilic molecules.^16,17^ Similar to polymers, the properties of LNP can be tuned by choosing different lipids and stabilizers or stabilizer mixtures, but in contrast to polymers, these small-molecule building blocks can be excreted *via* the kidneys.^18,19^ LNP formulations are becoming increasingly important in drug delivery not only due to their potential to deliver hydrophobic compounds within their core. They are further able to complex genetic material such as different types of RNA, if, for example, a potentially cationic stabilizer is added for complexation of the latter.^20^ Stabilizers can, depending on the nature of their hydrophilic, ionic, or ionizable head groups, further provide tissue or organ selectivity,^21^ regulate biocompatibility^13,22^ and release properties. Receptor-targeting molecules can be attached to enable accumulation of the particles in specific cell types or tissues, and the protein adsorption pattern of the particle can be influenced.^23^ The choice of the core-forming lipid, on the other hand, can influence particle stability. For example, a lipid with a melting point below body temperature can form a particle with a solid core and, therefore, higher stability (solid lipid nanoparticle, SLNP).^16^ If shorter degradation times are desired, choosing a liquid lipid may be of advantage.^24^ If the melting point of the lipid is close to body temperature, the stability of the particles may even change at a target location (temperature-responsiveness).^19^ Replacing the pure lipid with a lipid mixture in the case of solid lipid nanoparticles helps to prevent expulsion of the stabilizer, which can occur upon crystallization of the lipid.^25^ LNP sizes are dependent on both the stabilizer and the ratio of all compounds used in the formulation, and their internal structures can be complex, especially if genetic material is encapsulated.^20,26^

Modern LNP formulations that are used for, e.g., the encapsulation of mRNA-based vaccines, are comprised of a tailor-made mixture of lipids and stabilizers.^27,28^ Ionic or ionizable stabilizers and phospholipids are needed for particle integrity and encapsulation of the payload. The optimization of these small-molecule structures and the adjustment of their ratio within the LNP formulation has led to the discovery of selective organ targeting (SORT) mechanisms that allow for the delivery of LNP to a specific tissue (e.g., lung, liver or spleen).^21,27^

The second class of lipid stabilizers that are frequently used in LNP formulations are PEO-based lipids that prevent particle aggregation as well as opsonization.^29^ PEO was long time considered as the gold standard for polymers that adsorb a specific pattern of proteins *in vivo* leading to a low recognition of drug delivery vehicles with a PEO shell by the immune system.^23^ Nevertheless, *anti*-PEO antibodies were found in an increasing number of individuals in the last years, which may be attributed to the extensive use of PEO in drug formulations, medical, and cosmetical products.^30,31^ Therefore, the (partial) replacement of PEO with suitable polymeric alternatives^32−37^ in LNP formulations and medicinal chemistry beyond is required.

Poly(2-oxazoline)s have received a lot of attention in recent years due to their high biocompatibility and pharmacological properties that are similar or even more favorable than those of PEO.^36,38,39^ Further, they can be synthesized with narrow dispersities in a wide range of molecular weights by living cationic ring opening polymerization (CROP), and their properties can be tuned by the choice of the substituent in the 2-position of the monomer, which makes them more versatile than PEO.^40,41^ Functional groups that are incompatible with CROP can be protected during polymerization,^42,43^ or be introduced by (partial) hydrolysis of poly(2-methyl)- or poly(2-ethyl-2-oxazoline) and subsequent postpolymerization functionalization of the resulting secondary amino groups with the functional groups of choice *via* amide coupling reactions.^44−46^ Further, the usage of (multi)functional initiators and terminating nucleophiles can introduce additional functional groups to attach functional molecules such as dyes and targeting or drug molecules.^47−49^ First studies demonstrate that replacing PEO-based lipid stabilizers with hydrophilic poly(2-oxazoline)-based ones that contain 2-ethyl- or 2-methyl-2-oxazoline units and exhibit a similar degree of polymerization (DP) yields nontoxic, and nonimmunogenic LNPs that may even show superior performance when it comes to, e.g., biodistribution or endosomal escape of the particles.^28,50^ Those advantages inspired us to generate an LNP drug delivery system using poly(2-oxazoline)-based lipid stabilizers to target the passage of (solid) lipid nanoparticles through the blood–brain barrier (BBB).

While no specific SORT lipid stabilizers that target the brain are known, several methods to enable the passage of drug delivery systems to the brain are reported in literature. Besides the fact that LNP may pass the BBB *via* interactions with lipoprotein transporters,^51^ the installation of carbohydrate molecules to a drug delivery vehicle can facilitate the passage of the respective particles through the endothelium.^52^ The glucose transporter GLUT1 is abundant in (brain) endothelial cells, and glucose-functionalized nanoparticles exhibit increased uptake into those cells under certain clinical conditions (e.g., glycaemic control).^53^ Especially the hydroxyl groups in the 1-, 3-, and 4-position of glucose are important for the interaction between GLUT1 and glucose, which is why the attachment of the latter to a drug delivery vehicle *via* the 6-position is beneficial.^54,55^ In addition to possible interactions with GLUT transporters, glycopolymers can interact with the glycocalyx, a layer of carbohydrate-containing molecules on the cell surface.^56−58^ This presents another possibility to target specific cells beyond the BBB.^59^ As glycopolymers are biocompatible and hydrophilic, they also serve as a suitable polymer class for the hydrophilic part of a lipopolymer used for the formation of micelles or stabilization of LNP.^43,57,58^

In this manuscript, we present the synthesis of glucosylated, multifunctional poly(2-oxazoline)-based stabilizers for LNP *via* CROP of 2-ethyl-2-oxazoline, subsequent partial hydrolysis, and introduction of different amounts of glucose units *via* amide coupling postpolymerization modification. We used *tert*-butyl bromoacetate as initiator and *N*-(2-azidoethyl)octadecan-1-amine as termination reagent in CROP, thereby introducing a reactive carboxylic group at one and a hydrophobic anchor with a neighboring linker for the attachment of a fluorescent dye at the other end. As the glucose moieties are attached as side groups of the polymer instead of being attached at the end of the polymer chain, an additional bioactive molecule can be attached to the terminal carboxylic acid function, which potentially allows addressing both GLUT1 and a second target of choice. Thereby, we present the first attempt to combine the advantages of poly(2-oxazoline)s, glycopolymers, and solid lipid nanoparticles (SLNP) with a tetradecan-1-ol/cholesterol core as potential cell-specific drug delivery system. The presented polymers and SLNP showed low toxicity and interacted with PC3 and MDA-MB231 cells. While polymer–cell interactions were mainly dependent on the physicochemical properties of the polymer, especially the impact of the hydrophobic compartment, the stability of SLNP and their affinity toward the investigated cell lines was dependent on the functionalization of the stabilizing polymers with glucose. This may be attributed to glucose-related polymer–cell interactions, and the masking of the hydrophobic compartment of the polymers within the core of the SLNP. The presented polymeric stabilizers can be of interest for the generation of polymer-drug conjugates with cell membrane affinity^60^ or as stabilizers for LNP for the delivery of hydrophobic drugs. Our study will therefore contribute to current research on stabilizer-directed selective tissue targeting using LNP.

## 2 Experimental Section

### 2.1 Materials and Methods

Dry solvents in septum bottles were purchased from Sigma-Aldrich Ltd. (Prague, Czech Republic). All other solvents as well as sulfuric acid, HCl (aq), acetic acid, acetic anhydride, NaOH, KOH, and Na_2_SO_4_ were purchased from Lachner Ltd. (Neratovice, Czech Republic) and were of analytical grade. d(+)-glucose and paraformaldehyde were purchased from Carl Roth GmbH + Co. KG (Karlsruhe, Germany), and EDC hydrochloride was purchased from Carbolution Chemicals GmbH (St. Ingbert, Germany). The origin of chemicals used in bioexperiments is specified in Section 2.3. All other chemicals were purchased from Sigma-Aldrich Ltd. (Prague, Czech Republic). Chlorobenzene, 2-ethyl-2-oxazoline, and *tert*-butyl bromoacetate used in the synthesis of the polymers were dried over CaH_2_ or P_2_O_5_ under argon, distilled and stored over 4 Å molecular sieves prior to use. All other chemicals were used as received.

Sephadex-LH20 was purchased from Cytiva *via* Sigma-Aldrich Ltd. (Prague, Czech Republic), and equilibrated in methanol (MeOH) for 3 h before packing in a gravity-driven separation column.

Chloroform-d, MeOD, and DMSO-*d*_6_ were purchased from Eurisotop (Cambridge, U.K.).

Proton nuclear magnetic resonance (^1^H NMR) measurements were performed on a 400 MHz Bruker Avance Neo spectrometer using CDCl_3_ MeOD, or DMSO-*d*_6_ as a deuterated solvent. For calibration, the specific signals of the nondeuterated species were used.

Electron spray ionization mass spectrometry (ESI-MS) was carried out on a LCQ Fleet hybrid mass spectrometer (Thermo Fisher Scientific, Waltham, USA) equipped with an LTQ Orbitrap XL using methanol as mobile phase (flow rate 10 μL min^–1^) in positive mode. The data was processed with the Xcalibur Software (Thermo Fisher Scientific).

Matrix-assisted laser desorption ionization–time-of-flight mass spectrometry (MALDI-TOF MS) mass spectra were acquired with the UltrafleXtreme TOF – TOF mass spectrometer (Bruker Daltonics, Bremen, Germany) equipped with a 2000 Hz smartbeam-II laser (355 nm) using the positive ion linear mode. Panoramic pulsed ion extraction and external calibration were used for molecular weight assignment. The dried droplet method was used in which solutions of the sample (20 mg mL^–1^), the matrix (DHB, 2,5-dihydroxybenzoic acid, 20 mg mL^–1^), and the ionizing agent sodium trifluoroacetate (10 mg mL^–1^) in methanol are mixed in the volume ratio 4:20:1. One μL of the mixture was deposited on the ground-steel target.

Gel permeation chromatography (GPC) measurements in MeOH/acetate buffer were carried out on a Dionex UltiMate 3000 UHPLC chromatograph (ThermoFisher Sci, USA) equipped with an autosampler, an UV–VIS detector (323 nm), an Optilab rEX differential refractometer and a DAWN 8+ multiangle light scattering (MALS) detector (Wyatt; Santa Barbara, CA, USA). A TSK SuperAW3000 column with methanol and sodium acetate buffer (pH = 6, 8:2 v/v) as an eluent at a flow rate of 0.5 mL min^–1^ was used.

Gel permeation chromatography (GPC) measurements in DMSO were performed using a DeltaChrom SDS 030 pump (Watrex Ltd., Czech Republic) with a flow rate of 0.5 mL min^–1^. The two PLgel 10 μm mixed B LS columns (Polymer Laboratories, UK, separation range of approximately 5 × 102 ≤ M ≤ 1 × 107 as determined using PS standards) were used in a series. A DAWN HELEOS II MALS detector (Wyatt Technology Corp., Germany) with a laser operating at a wavelength λ = 658 nm, and an Optilab T-rEX RI detector (Wyatt Technology Corp., Germany) were used. Dimethyl sulfoxide (≥99%, HPLC grade, Fisher Scientific, Czech Republic) with 0.05 M LiBr (≥99%, Merck, Czech Republic) as an additive was used as the mobile phase at ambient temperature. The sample injection volume was 100 μL. The data was collected using the Astra software (Wyatt Technology Corp.). *M*_w_ and *M*_n_ were calculated with a d*n*/d*c* = 0.15.

Dynamic light scattering (DLS) measurements of the polymers and SLNP in water were performed on an ALV-6010 SLS/DLS instrument (ALV-GmbH, Germany) equipped with a 22 mW He–Ne laser (λ = 632.8 nm) at a detection angle of 90°. Measurements were carried out at 25 °C. Solvent viscosity and refractive index were automatically adjusted to the temperature of the thermostat. The CONTIN algorithm was applied to analyze the obtained correlation functions. Apparent hydrodynamic radii were calculated according to the Stokes–Einstein equation
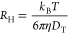
where *R*_H_ is the hydrodynamic radius, *k*_B_ is the Boltzmann constant, *T* is the absolute temperature, η is the dynamic viscosity of the solvent, and *D*_T_ is the translational diffusion coefficient.

DLS measurements of the polymers and SLNP in DMEM at 37 °C were carried out on a Nano-ZS Zetasizer ZEN3600 (Malvern Instruments, UK). DMEM was filtered with a 0.22 μM filter prior to use.

Fluorescence measurements for the determination of the critical micelle concentration were carried out on a Multimode Microplate Reader (BioTek Synergy H1, Agilent, US). A stock solution of Nile Red in DMEM (0.12 mmol) was prepared by mixing 4 μL of a Nile Red solution (3.1 mmol in THF) with 100 mL of DMEM. The 2 mg mL^–1^ stock solutions of the polymers in DMEM were diluted with pure DMEM and DMEM containing Nile Red to obtain solutions with a concentration of 1, 0.5, 0.25, 0.05, and 0.01 mg mL^–1^, respectively. The fluorescence of the Nile Red-containing solutions was measured on a 96-well plate with an excitation wavelength of 515 nm and an emission wavelength of 585 nm.

High-resolution scanning transmission electron microscopy (STEM) imaging of stabilized solid lipid nanoparticles was performed on a custom-modified Quanta 650 FEG environmental scanning electron microscope (ESEM) (Thermo Fisher Scientific, MA, USA) equipped with a scanning transmission electron microscopy (STEM) detector.^61^ The samples, dissolved in distilled water, were applied to a lacey carbon film on a copper TEM grid.^62^ Then, the samples were in situ freeze-dried at −20 °C and 10 Pa in the ESEM specimen chamber (operated under environmental mode). Observation was performed at a beam energy of 30 keV, a beam current of 5 pA, and a working distance of 5.3 mm in high vacuum mode using a dark field STEM detector. The micrographs were postprocessed using MountainsSEM software (Digital Surf, France). Particle sizes were measured using ImageJ software.

Fourier transform infrared (FTIR) spectra were measured on a Spectrum 100T FT-IR spectrometer (PerkinElmer, USA) equipped with a deuterated triglycine sulfate detector using the attenuated total reflectance (ATR) technique. Four scans per spectrum (650–4000 cm^–1^) at the resolution of 4 cm^–1^ were measured.

UV/vis spectra of the cyanine 3/cyanine 5 (cy3/cy5)-labeled compounds were recorded on an Evolution 220 UV/vis spectrometer (Thermo Scientific, USA) using solutions of the compounds in micropure water (10 μg mL^–1^).

Fluorescence correlation spectroscopy (FCS) uses time change in fluctuations of fluorescence intensity to obtain separate FCS autocorrelation functions (ACFs) of individual fluorophore populations in a mixture. We used this technique to probe the presence of different fluorescently labeled species in our polymer and LNP solutions. The samples were diluted to obtain reasonable concentrations of the fluorophore within the observation confocal volume (200× in case of the LNP, and 600× in case of the polymer solution, starting from solutions based on 1 mg mL^–1^ polymer) and subsequently excited by an LDH-D-C-640 laser diode emitting 640 nm light, driven by a PDL 828 Sepia II driver in picosecond pulsed mode at a 20 MHz repetition rate (both devices: PicoQuant) through the 635 nm dichroic mirror built into the IX83 scan head. An Olympus UPlanSApo water immersion objective (60×, 1.2 NA) delivered the excitation light into a diffraction-limited spot and collected the emitted fluorescence. The laser intensity was maintained at approximately 10 μW average power at the objective entrance pupil to avoid photobleaching and/or saturation. The collected fluorescence light passed through a Semrock 690/70 nm BrightLine emission filter and was detected by a hybrid photomultiplier (PMA Hybrid-40 from PicoQuant) operated in photon counting mode. Photon counts were recorded using a PicoHarp300 TCSPC module in a T3 time tagging mode. The SymPhoTime64, ver. 2.1 software from PicoQuant was used for data acquisition and FCS data analysis. Each acquisition took 1 min, and the measurements were performed at 23 ± 1 °C. The FCS autocorrelation function (ACF) for the simplest case of one diffusing component is mathematically given by equation
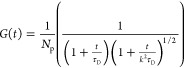
wherein *N*_p_ is the average number of diffusing fluorescent particles in the confocal volume, *t* is the correlation time, the diffusion time τ_D_ refers to the residence time of fluorescent objects in focus and *k* is the ratio of axial to radial radii of the confocal volume, *k* = *w*_*z*_*/w*_*xy*_ with *w*_*xy*_ and *w*_*z*_ being the dimensions of the focal spot in the *x*–*y* plane (perpendicular to the optical axis) and along the *z*-axis. Then, the diffusion time can be expressed as τ_D_*= w*^2^_*xy*_*/*4*D*_T_, where *D*_T_ is the coefficient of translational diffusion of particles. Diffusion coefficients were obtained by fitting of measured ACFs with appropriate model functions and hydrodynamic radii of the polymers and LNP in aqueous solution were subsequently obtained using the Stokes–Einstein equation
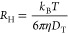
where *R*_H_ is the hydrodynamic radius, *k*_B_ is the Boltzmann constant, *T* is the absolute temperature, η is the dynamic viscosity of the solvent, and *D*_T_ is the translational diffusion coefficient.

### 2.2 Synthesis

#### 2.2.1 Synthesis of Small-Molecular Weight Compounds

##### 2.2.1.1 6-Acetyl-1:2–3:4-bismethylene-d-glucofuranose^63^

D-(+)-Glucose (10 g, 55.5 mmol) was dissolved in 4 mL of distilled water in a round-bottom flask. Then, 40 mL of glacial acetic acid and paraformaldehyde (11 g, 366 mmol formaldehyde) were added. The flask was immersed in an oil bath and gradually heated to 80 °C while 5 mL of concentrated sulfuric acid were added dropwise. Stirring at 80 °C was continued for 1 h before 40 mL of cold, distilled water were added. After cooling to room temperature, the mixture was extracted with chloroform (50 mL, 3×). The combined organic phases were washed with brine (1×), dried over Na_2_SO_4_, filtered, and concentrated under reduced pressure. The crude product was obtained as a yellow, viscous oil and purified *via* flash column chromatography on silica gel (cyclohexane/ethyl acetate 2:1) to obtain 4.5 g (18.3 mmol, 33%) of the pure product as white crystals.

^1^H NMR (400 MHz, CDCl_3_, δ): = 6.18 (d, *J* = 3.7 Hz, 1H), 5.23 (s, 1H), 5.17 (s, 1H), 5.07 (d, *J* = 6.3 Hz, 1H), 4.95 (d, *J* = 5.8, 1H), 4.64 (d, *J* = 3.7 Hz, 1H), 4.62 (d, *J* = 3.7 Hz, 1H), 4.59 (d, *J* = 6.4 Hz, 1H), 4.45–4.32 (m, 3H), 4.09–4.06 (t, *J* = 1.4 Hz, 1H), 2.25 (s, 3H).

##### 2.2.1.2 1:2–3:4-Bismethylene-d-glucofuranose^63^

6-Acetyl-1:2–3:4-bismethylene-d-glucofuranose (4.5 g, 18.3 mmol) was dissolved in 40 mL of dry methanol in a round-bottom flask under argon and cooled to 0 °C. Then, sodium methoxide (5.4 M in MeOH, 3.6 mL, mmol) was added slowly to the solution. The mixture was stirred at 0 °C for 1 h and the solvent was subsequently removed under reduced pressure. The residue was suspended in dichloromethane, the solid residue was filtered off and washed with additional solvent. The solvent was then removed under reduced pressure, and the resulting transparent oil was dried under high vacuum to yield 3.5 g (17.1 mmol, 94%) of the desired product in sufficient purity for the next step.

^1^H NMR (400 MHz, CDCl_3_, δ): = 6.16 (d, *J* = 3.8 Hz, 1H), 5.20 (s, 1H), 5.16 (s, 1H), 5.11 (d, *J* = 5.9 Hz, 1H), 4.97 (d, *J* = 5.9 Hz, 1H), 4.63 (d, *J* = 3.7 Hz, 1H), 4.43 (d, *J* = 2.6 Hz, 1H), 4.23 (ddt, *J* = 8.9, 5.9, 3.0 Hz, 1H), 4.13 (t, *J* = 2.4 Hz, 1H), 4.04 (dd, *J* = 9.1, 6.4 Hz, 1H), 3.95 (dd, *J* = 11.7, 4.5 Hz, 1H).

##### 2.2.1.3 1:2–3:4-Bismethylene-d-glucofuranose-6-propanoic acid methyl ester^64^

1:2–3:4-Bismethylene-d-glucofuranose (2 g, 9.77 mmol) was dissolved in 50 mL of DCM. Then, tetrabutylammonium bromide (TBABr, 630 mg, 1.95 mmol), methyl acrylate (5 mL, 61 mmol), and 5 mL of a saturated solution of sodium hydroxide in water were added. The reaction vessel was sealed, the mixture was degassed with argon and stirred at room temperature for 24 h. Afterward, brine (10 mL) was added to the mixture, the phases were separated, and the aqueous phase was washed with DCM (20 mL, 2×). The combined organic phases were dried over Na_2_SO_4_, filtered, and concentrated under reduced pressure to yield a mixture of the crude product and TBABr, which was purified *via* flash column chromatography on silica gel (cyclohexane/ethyl acetate 2:1) to obtain 2.02 g (6.96 mmol, 71%) of the pure product as colorless oil.

^1^H NMR (400 MHz, CDCl_3_, δ): = 6.13 (d, *J* = 3.8 Hz, 1H), 5.20 (s, 1H), 5.19 (d, *J* = 6.6 Hz, 1H), 5.14 (s, 1H), 4.88 (d, *J* = 6.0 Hz, 1H), 4.57 (d, *J* = 3.7 Hz, 1H), 4.41 (d, *J* = 2.3 Hz, 1H), 4.23 (t, *J* = 3.9 Hz, 1H), 3.95 (dd, *J* = 10.4, 3.5 Hz, 1H), 3.92–3.76 (m, 4H), 3.81 (s, 3H), 2.70 (t, *J* = 6.1 Hz, 2H).

##### 2.2.1.4 1:2–3:4-Bismethylene-d-glucofuranose-6-propanoic acid

1:2–3:4-Bismethylene-d-glucofuranose-6-propanoic acid methyl ester (1.85 g, 6.36 mmol) was dissolved in 60 mL of MeOH. Sodium hydroxide (1.8 g, 45 mmol) was dissolved in 15 mL of distilled water to obtain a 3 M aqueous solution that was added to the mixture. The reaction vessel was sealed, and the mixture was heated to 60 °C for 1 h. After cooling the reaction mixture to room temperature, it was neutralized by dropwise addition of concentrated HCl. Then, the solvents were evaporated under reduced pressure and the residue was suspended in DCM. The insoluble salts were removed *via* filtration and washed with DCM. The residue was dried over Na_2_SO_4_, filtered, and the solvent was removed under reduced pressure to yield 1.75 mg (3.17 mmol, 99%) of the desired product in sufficient purity as a colorless oil.

^1^H NMR (400 MHz, CDCl_3_, δ): = 9.17 (s, br, 1H), 6.16 (d, *J* = 3.7 Hz, 1H), 5.22 (s, 1H), 5.22 (d, *J* = 5.9 Hz, 1H) 5.16 (s, 1H), 4.92 (d, *J* = 5.9 Hz, 1H), 4.59 (d, *J* = 3.7 Hz, 1H), 4.49 (d, *J* = 2.3 Hz, 1H), 4.29 (t, *J* = 3.9 Hz, 1H), 4.14 (s, 1H), 3.98 (dd, *J* = 10.4, 3.2 Hz, 1H), 3.95–3.80 (m, 3H), 2.73 (t, *J* = 6.0 Hz, 2H).

##### 2.2.1.5 1,2,3,4-Tetra-*O*-acetyl-d-glucose-6-propanoic acid^63,65^

1:2–3:4-Bismethylene-d-glucofuranose-6-propanoic acid (1.6 g, 5.80 mmol) was dissolved in 72 mL of distilled water in a round-bottom flask equipped with a reflux condenser. Sixteen mL of concentrated HCl were added, and the mixture was heated at reflux for 3 h. Then, the mixture was allowed to cool to room temperature and the solvent was removed under reduced pressure. To remove traces of water, pyridine was added to the resulting light-yellow oil and removed together with the water under reduced pressure. Subsequently, the residue was dissolved in 60 mL of dry pyridine and the vessel was degassed with argon. The solution was then cooled to 0 °C and 4-dimethylaminopyridine (DMAP, 400 mg, 3.27 mmol) and 20 mL of acetic anhydride were added. Stirring was continued for 18 h while the mixture was allowed to reach room temperature. Afterward, the solvent was removed under reduced pressure to obtain 2.3 g of a mixture of the crude product and DMAP. Purification was realized by flash column chromatography on silica gel (cyclohexane/ethyl acetate/TFA 1:1:0.002) to obtain 1.24 g (2.95 mmol, 51% over both steps) of the pure product as a white solid. The product contains a mixture of the α- and β-glucose derivative in a ratio of 44:56.

^1^H NMR (400 MHz, CDCl_3_, δ): = 7.88 (s, br, 1H α, 1H β), 6.45 (d, *J* = 3.7 Hz, 1H, α), 5.82 (d, *J* = 8.3 Hz, 1H, β), 5.59 (t, *J* = 9.6 Hz, 1H, α), 5.37 (t, *J* = 9.4 Hz, 1H, β), 5.33–5.17 (m, 2H α, 2H β), 4.17 (ddd, *J* = 10.3, 4.2, 2.7 Hz, 1H, α), 3.96–3.64 (m, 4H α, 5H β), 2.76 (t, *J* = 5.2 Hz, 2H α, 2H β), 2.31, 2.24, 2.18, 2.17, 2.16, 2.16, 2.15, 2.15 (multiple s, 12H α, 12H β).

##### 2.2.1.6 2-(Octadecylamino)ethan-1-ol^66^

Ethanolamine (1.43 mL, 23.4 mmol), bromooctadecane (4 mL, 11.7 mmol), and triethylamine (1.8 mL, 12.9 mmol) were dissolved in 60 mL of ethanol, degassed, and stirred at 85 °C overnight. Then, the mixture was allowed to cool to room temperature, the solvent was evaporated under reduced pressure and the residue was taken up in DCM. The solution was washed with water (1×) and brine (2×), dried over Na_2_SO_4_, filtered, and concentrated to yield 3.5 g (11.2 mmol, 95%) of the crude product as a white solid. The crude product can be recrystallized from ethyl acetate (Yield: 2.2 g, 7.0 mmol, 60%).

^1^H NMR (400 MHz, CDCl_3_, δ): = 3.77 (t, *J* = 6.9 Hz, 2H), 2.91 (m, *J* = 5.2 Hz, 2H), 2.75 (m, *J* = 7.2 Hz, 2H), 1.61 (quint, *J* = 6.8 Hz, 2H), 1.50–1.32 (m, 32H), 1.01 (t, *J* = 6.9 Hz, 3H).

ESI–MS: found: *m*/*z* = 313.008, calculated: *m*/*z* = 314.342 [M + H^+^].

##### 2.2.1.7 *N*-(2-chloroethyl)octadecan-1-amine hydrochloride^67^

2-(Octadecylamino)ethan-1-ol (1.0 g, 3.2 mmol) was dissolved in 12 mL of dry chloroform, degassed, and cooled to 0 °C. Then, a solution of thionyl chloride (1.122 mL, 15.4 mmol) in 3 mL of dry chloroform was added slowly to the mixture. The reaction vessel was allowed to reach room temperature during the next 30 min and was subsequently heated to 65 °C for 6 h. Then, the mixture was cooled to room temperature again and 1 mL of methanol was added carefully to quench residual thionyl chloride. The solvents were removed from the mixture under reduced pressure and the residue was washed with warm ethyl acetate to yield 1.01 g (2.7 mmol, 86%) of the product as an off-white solid.

^1^H NMR (400 MHz, MeOD, δ): = 4.03 (t, *J* = 6.0 Hz, 2H), 3.56 (t, *J* = 6.0 Hz, 2H), 3.20 (dd, *J* = 9.0, 7.0 Hz, 2H), 1.87 (quint, *J* = 7.6 Hz, 2H), 1.63–1.35 (m, 32H), 1.03 (t, *J* = 6.8 Hz, 3H).

ESI–MS: found: *m*/*z* = 331.000, calculated: *m*/*z* = 332.308 [M + H^+^].

##### 2.2.1.8 *N*-(2-azidoethyl)octadecan-1-amine^67^

*N*-(2-chloroethyl)octadecan-1-amine hydrochloride (1.01 g, 2.7 mmol) was suspended in 15 mL of dry DMF under argon. Then, solid sodium azide (750 mg, 11.5 mmol) was added to the mixture, the vessel was sealed, and the mixture was stirred at 80 °C for 18 h. Afterward, the solvent was removed under reduced pressure, the residue was taken up in DCM and the remaining salts were filtered off and washed with additional DCM. Despite the fact that sodium azide is not soluble in cold DCM and was therefore not expected to react with the solvent, other organic solvents such as diethyl ether may be preferred for the washing step to reduce the risk of forming explosive diazidomethane.^68^ Evaporation of the solvent yielded 770 mg of an orange solid. The crude product was purified *via* flash column chromatography on silica gel (cyclohexane/ethyl acetate/triethylamine 8:2:0.02) to obtain 450 mg (1.33 mmol, 49%) of the pure product as a light-yellow solid.

^1^H NMR (400 MHz, CDCl_3_, δ): = 3.55 (m, 2H), 2.92 (m, 2H), 2.74 (t, *J* = 7.2 Hz, 2H), 1.61 (quint, *J* = 6.8 Hz, 2H), 1.50–1.30 (m, 32H), 1.01 (t, *J* = 6.9 Hz, 3H).

ESI–MS: found: *m*/*z* = 336.812, calculated: *m*/*z* = 339.348 [M + H^+^].

##### 2.2.1.9 Cholesterol-*O*-propanenitrile^69^

Cholesterol (1.544 g, 4 mmol) and 18-crown-6 (104 mg, 0.4 mmol) were dissolved in 30 mL of DCM. Then, 1 mL of a 40 wt % solution of potassium hydroxide was added, the mixture was degassed, and acrylonitrile (2.6 mL, 40 mmol) was added. The solution was stirred at room temperature for 18 h. Then, the mixture was washed with water (30 mL, 1×) and brine (30 mL, 2×), and dried over Na_2_SO_4_. The solid was filtered off and the filtrate was concentrated under reduced pressure to yield ca. 2.5 g of a yellow solid that was purified *via* flash column chromatography (cyclohexane/diethyl ether 9:1) to yield the pure product (1.55 g, 3.53 mmol, 88%) as a white solid.

^1^H NMR (400 MHz, CDCl_3_, δ): = 5.49 (dd, *J* = 3.2, 2.1 Hz, 1H), 3.83 (t, *J* = 6.4 Hz, 2H), 3.35 (tt, *J* = 11.3, 4.5 Hz, 1H), 2.71 (t, *J* = 6.5 Hz, 2H), 2.53–2.44 (m, 1H), 2.42–2.29 (m, 1H), 2.20–1.88 (m, 5H), 1.78–1.06 (m, 21H), 1.13 (s, 3H), 1.05 (d, *J* = 6.6 Hz, 3H), 1.00 (dd, *J* = 6.6, 1.8 Hz, 6H), 0.81 (s, 3H).

##### 2.2.1.10 Cholesterol-*O*-(*N*-Boc-propan-1-amine)^69^

Cholesterol-*O*-propanenitrile (1.2 g, 2.73 mmol) was dissolved in 30 mL of THF. Then, a solution of 740 mg (3.4 mmol) of Boc anhydride and 650 mg (2.8 mmol) of NiCl_2_ hexahydrate in 60 mL of methanol was added, the mixture was degassed with argon and cooled to 0 °C in an ice bath. NaBH_4_ (740 mg, 19.6 mmol) was added portion wise as a solid. The formation of a gas and of a fine, black precipitate was observed. The mixture is stirred for 18 h while being allowed to reach room temperature. Then, the solvents were evaporated under reduced pressure, and the residue was taken up in ethyl acetate (100 mL). The solution was washed with brine (80 mL, 3×), the organic phase was dried over Na_2_SO_4_, the solids were filtered off, and the solution was concentrated under reduced pressure to yield ca. 2 g of the crude product as an off-white solid. Purification was carried out *via* flash column chromatography (cyclohexane/diethyl ether 9:1) to yield 1.15 mg (2.11 mmol, 77%) of the pure product as a white solid.

^1^H NMR (400 MHz, CDCl_3_, δ): = 5.48 49 (dd, *J* = 3.2, 2.1 Hz, 1H), 5.05 (s, br, 1H), 3.67 (t, *J* = 6.4 Hz, 2H), 3.36 (dd, *J* = 11.8, 5.8 Hz, 2H), 3.27 (tt, *J* = 11.2, 4.4 Hz, 1H), 2.49 (ddd, *J* = 13.2, 4.7, 2.2 Hz, 1H), 2.39–2.24 (m, 1H), 2.22–1.92 (m, 5H), 1.87 (quint, *J* = 6.4 Hz, 2H), 1.75–1.06 (m, 21H), 1.58 (s, 9H), 1.14 (s, 4H), 1.05 (d, *J* = 6.6 Hz, 3H), 1.00 (dd, *J* = 6.6, 1.8 Hz, 6H), 0.81 (s, 3H).

##### 2.2.1.11 Cholesterol-*O*-propan-1-amine^69^

Cholesterol-*O*-(*N*-Boc-propan-1-amine) (1.15 g, 2.11 mmol) was dissolved in 6 mL of DCM, the solution was degassed and cooled to 0 °C in an ice bath. Then, 6 mL of TFA were added, and stirring was continued for 1 h. The solvents were removed under reduced pressure, the residue was taken up in a mixture of DCM and methanol (1:1), and the solution was neutralized by addition of aqueous ammonia. The solvents were removed, and the crude product was purified *via* flash column chromatography (DCM/MeOH/NH_4_OH (aq) 95:4.5:0.5) to yield 590 mg (1.33 mmol, 63%) of the pure product as a white solid.

^1^H NMR (400 MHz, CDCl_3_, δ): = 5.48 (dd, *J* = 3.0, 2.3 Hz, 1H), 3.70 (td, *J* = 6.2, 1.6 Hz, 2H), 3.27 (tt, *J* = 11.3, 4.4 Hz, 1H), 2.97 (t, *J* = 6.6 Hz, 2H), 2.49 (ddd, *J* = 13.1, 4.7, 2.1 Hz, 1H), 2.36–2.22 (m, 3H), 2.20–1.92 (m, 5H), 1.87 (quint, *J* = 6.4 Hz, 2H), 1.75–1.06 (m, 21H), 1.13 (s, 4H), 1.05 (d, *J* = 6.6 Hz, 4H), 1.00 (dd, *J* = 6.6, 1.8 Hz, 6H), 0.81 (s, 3H).

##### 2.2.1.12 Cy3-NHS Ester^70,71^

1,2,3,3-tetramethyl-3*H*-indol-1-ium iodide^60^ (100 mg, 0.33 mmol) and *N*,*N*′-diphenylformamidine (130 mg, 0.66 mmol) were dissolved in a mixture of AcOH and Ac_2_O (1:1, 3 mL). The mixture was stirred at 100 °C for 2 h. Then, the solvent was removed under reduced pressure, the residue was taken up in a small amount of DCM and precipitated in cold diethyl ether (20 mL). The precipitate was repeatedly washed with acetone to obtain the product (89 mg, 0.20 mmol, 60%) as a yellow solid.

^1^H NMR (400 MHz, CDCl_3_, δ): = 9.30 (d, *J* = 14.3 Hz, 1H), 7.83 (tt, *J* = 8.7, 1.8 Hz, 2H), 7.75 (ddt, *J* = 12.1, 10.9, 5.5 Hz, 1H), 7.71–7.56 (m, 6H), 5.69 (d, *J* = 14.3 Hz, 1H), 3.96 (s,fj 3H), 2.24 (s, 3H), 1.95 (s, 6H).

The product of the first step (80 mg, 0.18 mmol) and 6-(2,3,3-trimethyl-3*H*-indol-1-ium-1-yl)hexanoate^60^ (49 mg, 0.18 mmol) were dissolved in 3 mL of pyridine. The mixture was degassed, and stirred at 40 °C for 30 min. Then, the solvent was evaporated, and the crude product was purified *via* flash column chromatography (DCM/MeOH 95:5) to yield 46 mg (0.093 mmol, 52%) of the product as a pink solid.

^1^H NMR (400 MHz, CDCl_3_, δ): = 8.54 (t, *J* = 13.4 Hz, 1H), 7.60–7.46 (m, 4H), 7.44–7.36 (m, 2H), 7.28 (dd, *J* = 20.7, 8.0 Hz, 2H), 7.10 (d, *J* = 13.4 Hz, 1H), 4.28 (t, *J* = 7.6 Hz, 2H), 3.95 (s, 3H), 2.71 (t, *J* = 6.8 Hz, 2H), 2.23–2.11 (m, 2H), 2.09–1.92 (m, 4H), 1.86 (s, 6H), 1.85 (s, 6H).

The product of step 2 (20 mg, 0.043 mmol) was dissolved in 5 mL of dry DCM, and degassed. Then, the solution was cooled to 0 °C in an ice bath, and EDC hydrochloride (12.3 mg, 0.064 mmol), *N*-hydroxysuccinimide (7.4 mg, 0.064 mmol), and 4-DMAP (10.5 mg, 0.086 mmol) were added. The mixture was allowed to warm to room temperature while stirring for 18 h. The residue was purified *via* flash column chromatography (DCM/MeOH/NH_4_OH (aq) 95:4.5:0.5) to yield 7 mg (0.012 mmol, 28%) of the pure product as a pink solid.

^1^H NMR (400 MHz, CDCl_3_, δ): = 8.53 (*t*, *J* = 13.4 Hz, 1H), 7.58–7.43 (m, 6H), 7.37 (t, *J* = 7.5 Hz, 2H), 7.27 (dt, *J* = 19.9, 9.9 Hz, 2H), 4.40 (t, *J* = 7.4 Hz, 2H), 3.94 (s, 3H), 2.96 (s, 4H), 2.79 (t, *J* = 7.2 Hz, 2H), 2.12–1.94 (m, 6H), 1.84 (s, 6H), 1.83 (s, 6H).

##### 2.2.1.13 Cholesterol-*O*-(*N*-cy3-propan-1-amine)

Cholesterol-*O*-propan-1-amine (10 mg, 22.5 μmol) and cy3-NHS ester (6.9 mg, 11.9 μmol) were dissolved in 5 mL of dry chloroform. The solution was degassed, and 100 μL of DIPEA were added. The mixture was stirred at room temperature for 18 h. Then, the mixture was diluted with 15 mL of DCM, washed with 0.1 M HCl (aq, 20 mL, 1×) and brine (20 mL, 2×), dried over Na_2_SO_4_, filtered, and the solvent was removed under reduced pressure. The residue was purified *via* flash column chromatography (DCM/MeOH/NH_4_OH (aq) 95:4.5:0.5) to yield 5 mg (5.7 mmol, 48%) of the pure product as a pink solid.

^1^H NMR (400 MHz, CDCl_3_, δ): = 8.53 (*t*, *J* = 13.4 Hz, 1H), 7.60–7.45 (m, 4H), 7.44–7.35 (m, 2H), 7.26 (dd, *J* = 7.9, 5.3 Hz, 2H), 7.20 (d, *J* = 13.6 Hz, 1H), 7.08 (d, *J* = 13.2 Hz, 1H), 5.44 (t, *J* = 2.6 Hz, 1H), 4.27 (t, *J* = 7.6 Hz, 2H), 3.90 (s, *J* = 10.6 Hz, 3H), 3.64 (td, *J* = 6.0, 2.2 Hz, 2H), 3.44 (dd, *J* = 12.3, 6.6 Hz, 2H), 3.24 (tt, *J* = 11.3, 4.4 Hz, 1H), 2.47 (t, *J* = 7.4 Hz, 2H), 2.55–1.89 (m, 15H), 1.86 (s, 6H), 1.85 (s, 6H), 1.75–1.06 (m, 21H), 1.09 (s, 3H), 1.05 (d, *J* = 6.6 Hz, 3H), 1.00 (dd, *J* = 6.6, 1.8 Hz, 6H), 0.80 (s, 3H).

ESI–MS: found: *m/z* = 882.720, calculated: *m/z* = 882.687 [M+].

#### 2.2.2 Synthesis and Modification of Polymers

##### 2.2.2.1 Bifunctional Poly(2-ethyl-2-oxazoline) (HOOC-P(EtOx)-C_18_)^47,62^

2-Ethyl-2-oxazoline (1 mL) was dissolved in 2 mL of dry chlorobenzene in a microwave vial in a glovebox. Then, *tert*-butyl bromoacetate (29 μL, 0.196 mmol or 49 μL, 0.332 mmol, depending on the targeted molecular weight) was added, the vessel was sealed, and the mixture was placed in a preheated heating bath (100 °C) for 24 h. Then, a solution of *N*-(2-azidoethyl)octadecan-1-amine in dry chlorobenzene (100 mg mL^–1^, 1.3 mL, 0.393 mmol or 2.2 mL, 0.650 mmol, depending on the targeted molecular weight) was added and stirring at 100 °C was continued for 18 h. Afterward, the mixture was allowed to cool to room temperature, and the polymers were precipitated in 50 mL of cold diethyl ether, respectively. To remove residual end-capping reagent, the products were dialyzed against CHCl_3_/EtOH (1:1) using RC dialysis membranes (SpectraPor) with a molecular weight cutoff (MWCO) of 1000 g mol^–1^. Subsequent drying of the products yielded ca. 850 mg (0.170 or 0.283 mmol, respectively) of each polymer.

#### 2.2.3 Polymer P1

*M*_n_ (GPC): 3500 g mol^–1^, D̵ = 1.07; *M*_n_ (^1^H NMR): 3700 g mol^–1^, DP = 33; ^1^H NMR (400 MHz, MeOD, δ): = 4.33, 4.31, 4.24, 4.15 (multiple s, 2H), 3.89–3.47 (m, 128H), 2.85 (s, br, 2H), 2.71–2.42 (m, 68H), 1.63, 1.62, 1.60, 1.59 (multiple s, 9H), 1.57–1.37 (m, 34H), 1.31–1.16 (m, 96H), 1.04 (t, *J* = 6.9 Hz, 3H).

#### 2.2.4 Polymer P2

*M*_n_ (GPC): 6200 g mol^–1^, D̵ = 1.05; *M*_n_ (^1^H NMR): 5900 g mol^–1^, DP = 55; ^1^H NMR (400 MHz, MeOD, δ): = 4.33, 4.31, 4.25, 4.15 (multiple s, 2H), 3.80–3.52 (m, 220H), 2.84 (s, br, 2H), 2.69–2.42 (m, 114H), 1.63, 1.62, 1.60, 1.59 (multiple s, 9H), 1.51–1.40 (m, 34H), 1.32–1.17 (m, 165H), 1.03 (t, *J* = 6.9 Hz, 3H).

##### 2.2.4.1 Partial Deprotection of P1 and P2 to Bifunctional Poly(2-ethyl-2-oxazoline)-*co*-poly(ethylene imine) (HOOC-P(EtOx-*co*-EI)-C_18_)^44^

Small portions of P1 or P2 (50 mg, 8.3 μmol or 13.2 μmol, respectively, ca. 0.5 mmol of monomer units) were dissolved in 500 μL of micropure water in microwave vials. 500 μL of concentrated hydrochloric acid was added to each vial to obtain an aqueous solution of the polymer in 6 M HCl. The microwave vials were sealed and heated to 100 °C for 20–360 min. Then, the solutions were allowed to cool to room temperature and were neutralized with aqueous NaOH (6M). The neutral to basic solutions were freeze-dried and the solid residues, consisting of sodium chloride, sodium propionate and the polymeric product, were resuspended in methanol. The suspension was filtered through a paper filter and purified on a Sephadex LH20 column in methanol to obtain 17–45 mg of the pure products.

^1^H NMR (400 MHz, MeOD, δ): = 4.05 (s, 2H), 3.83–3.50 (m, 4H per EtO*x* unit), 3.17–2.70 (m, 4H per EI unit), 2.70–2.42 (m, 2H per EtOx unit), 1.61 (s, br, 2H), 1.49–1.39 (m, 32H), 1.25 (t, *J* = 7.4 Hz, 3H per EtOx unit), 1.04 (t, *J* = 6.9 Hz, 3H).

To obtain the polymers with 100% EtOx units and the same end group as the partially functionalized compounds (carboxylic acid instead of *tert*-butyl acetate), 50 mg (8.3 μmol or 13.2 μmol, respectively) of P1 or P2 were dissolved in a mixture of trifluoroacetic acid and micropure water (95:5) at 0 °C and stirred under argon for 1 h. Then, cooling was removed and stirring was continued for 2 h. Subsequently, all volatiles were removed, and the products were purified on a Sephadex LH20 column in methanol to obtain ca. 45 mg of the pure products. ^1^H NMR spectroscopy confirmed the absence of the *tert*-butyl protecting group.

The composition and molecular weight of all polymers is given in Table 1.

**Table 1:** Composition and Molecular Weight of all Polymers of the Formula HOOC-P(EtOx_n–x_-*co*-EI_x_)_n_-C_18_, Obtained *via* Partial Hydrolysis of Polymers P1 and P2

composition	reactant	ratio EtOx/EI (^1^H NMR)	*M*_n_ (^1^H NMR)
HOOC-P(EtOx_33_)-C_18_	P1	100:0	3700
HOOC-P(EtOx_29_-*co*-EI_4_)-C_18_	P1	88:12	3500
HOOC-P(EtOx_25_-*co*-EI_8_)-C_18_	P1	75:25	3300
HOOC-P(EtOx_13_-*co*-EI_20_)-C_18_	P1	40:60	2700
HOOC-P(EtOx_3_-*co*-EI_30_)-C_18_	P1	10:90	2100
HOOC-P(EtOx_1_-*co*-EI_32_)-C_18_	P1	3:97	1900
HOOC-P(EtOx_55_)-C_18_	P2	100:0	5800
HOOC-P(EtOx_49_-*co*-EI_6_)-C_18_	P2	90:10	5700
HOOC-P(EtOx_40_-*co*-EI_15_)-C_18_	P2	73:27	5200
HOOC-P(EtOx_25_-*co*-EI_30_)-C_18_	P2	45:55	4300
HOOC-P(EtOx_6_-*co*-EI_49_)-C_18_	P2	10:90	3200
HOOC-P(EtOx_1_-*co*-EI_54_)-C_18_	P2	3:97	2900

##### 2.2.4.2 Bifunctional Poly(2-ethyl-2-oxazoline)-*co*-poly(2-tetraacetylglucosyl-2-oxazoline) (HOOC-P(EtOx-*co*-AcGluOx)-C_18_)^45^

1,2,3,4-Tetra-*O*-acetyl-d-glucose-6-propanoic acid was dissolved in dry DMF at a concentration of 100 mg mL^–1^ (0.238 mmol mL^–1^). The stock solution was split into separate vials containing 20–110 mg of reactant, depending on the mass and degree of deprotection of the polymer to be functionalized. 1.2 equiv of reactant were used per unit of ethylene imine in the polymer. If the volume of the solution was lower than 1 mL, it was adjusted to 1 mL using dry DMF. The vials were sealed, degassed with argon, and 1.5 equiv of diisopropylethylamine and 1.2 equiv of PyBOP in DMF (100 mg mL^–1^, 0.192 mmol mL^–1^) were added. The mixture was stirred at room temperature for 5 min before a solution of the partially deprotected polymer P1 or P2 (12–20 mg in 1 mL of dry DMF) was added. Stirring was continued overnight at room temperature under argon. Then, the solvent was removed under reduced pressure and the residue was purified on a Sephadex LH20 column in methanol to yield 25–75 mg of the pure products.

^1^H NMR (400 MHz, MeOD, δ): = 6.42 (s, 1H per β-d-glucose unit), 5.95 (s, 1H per α-d-glucose unit), 5.56 (dd, *J* = 19.1, 10.0 Hz, 1H per β-glucose unit), 5.46 (s, 1H per α-glucose unit), 5.37–5.12 (m, *J* = 46.9 Hz, 2H per glucose unit), 4.38–3.51 (m, 9H per glucose unit, 4H per EtOx unit), 2.82 (s, 2H per glucose unit), 2.61 (s, 2H per EtOx unit), 2.41–2.04 (m, 12H per glucose unit), 1.58–1.37 (m, 32H), 1.25 (s, 3H per EtOx unit), 1.04 (t, *J* = 6.8 Hz, 3H).

##### 2.2.4.3 Deprotection to Bifunctional Poly(2-ethyl-2-oxazoline)-*co*-poly(2-glucosyl-2-oxazoline) (HOOC-P(EtOx-*co*-GluOx)-C_18_)

The acetyl protecting groups of HOOC-P(EtO*x*-*co*-AcGluO*x*)-C_18_ polymers were removed under basic conditions.^72,73^

For polymers P1–1, P1–2, P2–1, and P2–2, the polymer was dissolved in 3 mL of dry methanol under argon. Then, a catalytic amount of a 5.4 M solution of sodium methoxide in methanol was added. The mixture was stirred for 6 h at room temperature. The solution was neutralized by addition of Amberlite IR120 H^+^ ion-exchange resin. The resin was filtered off, and the products were purified on a Sephadex LH20 column in methanol to obtain 20–30 mg of the pure product.

^1^H NMR (400 MHz, MeOD, δ): = 5.08 (s, 1H per β-glucose unit), 4.45 (s, 1H per α-glucose unit), 4.08–3.10 (m, 4H per EtOx or GluOx polymer backbone unit, additional 8H per GluO*x* unit), 2.66 (s, 2H per GluOx unit), 2.56–2.27 (m, 2H per EtOx unit), 2.22–2.05 (m, 4H), 1.47 (s, 2H), 1.29 (s, 32H), 1.11 (t, *J* = 7.1 Hz, 3H per EtOx unit), 0.90 (t, *J* = 6.8 Hz, 3H).

For all other polymers, the respective compound was dissolved in 5 mL of micropure water, and 50 mg of solid NaOH were added to obtain a solution of the polymers in a 0.25 M solution of NaOH. The solutions were stirred at room temperature for 1 h. Then, the solution was neutralized by addition of Amberlite IR120 H^+^ ion-exchange resin. The resin was filtered off, and the products were purified on a Sephadex G25 column in micropure water to obtain 35–50 mg of the pure product.

^1^H NMR (400 MHz, DMSO-*d*_6_, δ): = 6.72 (s, 1H per β-glucose unit), 6.37 (s, 1H per α-glucose unit), 4.99, 4.80, 4.60, 4.38 (s, 4H per glucose unit, hydroxyl group protons), 4.05–3.30 (m, 4H per EtOx or GluOx polymer backbone unit, 7H per α-glucose unit, 6H per β-glucose unit), 3.24 (s, 1H per β-glucose unit), 3.11 (s, 1H per α-glucose unit), 3.01 (s, 1H per β-glucose unit), 2.62 (s, 2H per GluOx unit), 2.40 (s, 2H per EtOx unit), 2.11 (m, 4H), 1.49 (s, 2H), 1.35 (s, 32H), 1.08 (s, 3H per EtOx unit), 0.97 (t, *J* = 6.7 Hz, 3H).

##### 2.2.4.4 Attachment of a cy5 Dye to HOOC-P(EtOx-*co*-AcGluOx)-C_18_

A cy5 fluorescent dye was attached to the azide group of the polymers *via* CuAAC (copper-catalyzed azide–alkyne cycloaddition). For this purpose, cy5 carboxylic acid, prepared as described in an earlier publication, was coupled with propargyl amine in a first step:

Cy5 carboxylic acid^60^ (50 mg, 0.103 mmol) was dissolved in 5 mL of dry DCM under argon. Then, PyBOP (65 mg, 0.124 mmol) and propargyl amine (50 μL, 1.04 mmol) were added and the mixture was stirred at room temperature under argon overnight. The product was precipitated into cold Et_2_O, and the supernatant was discarded after centrifugation (7500 rpm, 10 min). Drying under high vacuum yielded 60 mg (0.090 mmol as hexafluorophosphate) of the product as blue solid.

MALDI–TOF MS: 520.320 g mol^–1^ [M^+^].

In a second step, the reactive dye was coupled to the azide group pf the polymers. For this purpose, 10 mg of each polymer was dissolved in 1.5 mL of dry DMSO, and the solutions were degassed with argon. Subsequently, CuSO_4_ × 5H_2_O (4 mg, 15.8 μmol) and sodium ascorbate (14 mg, 15.8 μmol) were added, and the mixtures were stirred for 10 min. Then, a solution of the cy5 alkyne (350 μL, 6.72 μmol, 10 mg mL^–1^ in dry degassed DMSO) was added to each mixture and stirring under argon was continued at room temperature overnight. Then, a solution of 11 mg (32.7 μmol) of EDTA disodium salt in 2 mL of micropure water was added, the mixture was transferred to a RC dialysis membrane (SpectraPor) with a molecular weight cutoff (MWCO) of 1000 g mol^–1^, and excess dye, reagents, and DMSO were removed *via* dialysis against a mixture of ethanol and water (1:1). After the dialysis, the polymers were additionally purified on a Sephadex LH20 with methanol (P1–0 – P1–2 and P2–0 – P2–2) or DMSO (P1–3 – P1–5 and P2–3 – P2–5) as an eluent. After solvent removal on rotary evaporator (methanol) or by freeze-drying (DMSO), the polymers were dissolved in water, eluted through a Sephadex G25 column in water, and freeze-dried.

#### 2.2.5 Preparation of Solid-Lipid Nanoparticles^19^

For the generation of nonlabeled, polymer-stabilized solid-lipid nanoparticles, 10 mg mL^–1^ stock solutions of tetradecan-1-ol and cholesterol in dichloromethane were prepared. Then, 30 μL (0.3 mg) of the tetradecan-1-ol and 10 μL (0.1 mg) of the cholesterol stock solutions were added to 200 μL of dichloromethane in a glass vial. The solution was homogenized by shaking and placed in a preheated oven at 50 °C. Polymer stock solutions (1 mg mL^–1^ in micropure water) were prepared separately and placed in the oven at 50 °C. After evaporation of dichloromethane, 1 mL of the desired polymer solution was added to the glass vial containing a melt of tetradecan-1-ol with dissolved cholesterol. The mixtures were homogenized by stirring at 8000 rpm for 1 min using a high-speed homogenizer system (T25 digital Ultra-Turrax, IKA, Schoeller instruments, s.r.o., Czech Republic) and subsequent exposition of the solution to ultrasound pulses (50% cycle) and a power output with an amplitude of 20% 1-RM for 10 min. The samples were allowed to cool down to room temperature prior to further investigation.

For stability measurements *via* DLS in DMEM at 37 °C, freshly prepared SLNPs with nonlabeled polymers were diluted with DMEM in a volume ratio of 1:9.

For the generation of fluorescently labeled, polymer-stabilized solid-lipid nanoparticles, 30 wt % of cholesterol in the cholesterol stock solution was replaced with cy3 labeled cholesterol. Further, 50 wt % of the nonlabeled polymer in the polymer stock solution was replaced with cy5 labeled polymer. Solid lipid nanoparticles were then prepared according to the procedure described above.

### 2.3 *In Vitro* Cell Assays

#### 2.3.1 Cell Lines

For the evaluation of polymer–nanoparticle interactions in biological systems, MDCK, PC3, and MDA-MBA-231 cell lines were selected based on information from literature. These cell lines were obtained as a kind gift from the group of Jan Konvalinka (Institute of Organic Chemistry and Biochemistry, Czech Academy of Sciences). The cell lines were maintained in DMEM or RPMI medium (Merck) supplemented with 4 mM glutamine (Gibco) and 10% fetal bovine serum (Gibco) at 37 °C in a 5% CO_2_ atmosphere. All analyses were performed with cell passages lower than 15.

#### 2.3.2 Normalization of Polymer and Nanoparticle Absorbance

The absorbance of 5 or 10 μM polymer and nanoparticle solutions in distilled water was measured on a Helios alfa spectrophotometer (Unicam). For the measurement, 1 mL of the solution was transferred to a cuvette and measured against a blank in the range 400–700 nm using the Vision 32 software. The signal of Cy3 and Cy5 was determined as absorbance at 552 and 650 nm, respectively. The degree of functionalization was then calculated using the tabulated extinction coefficient of 150000 for Cy3 and 250000 for Cy5.

#### 2.3.3 Cell Viability

Two thousand MDCK cells in 100 μL of medium were plated into each well of a 96-well plate. After an incubation period of 24 h after cell seeding, the polymers were added to the growth medium to achieve the target concentration in a range between 2.44 nM to 10 μM. Each concentration was tested in triplicate. The plates containing the cells were then incubated for 48 h at 37 °C in an atmosphere containing 5% CO_2_ in an automated Biospa incubator (Agilent Biotek), which allowed automatic cell counting using an automated Cytation 5 microscope (Agilent Biotek). The number of cells in each well was analyzed every 8 h and the final cell count ratio was analyzed against the control to assess toxicity. The data were then analyzed using the GraphPad Prism software to generate a dose-dependent viability curve using a sigmoid versus normalized response fit, with each data point measured at least in triplicate.

#### 2.3.4 MTT Viability Assay with Polymers

For each cell line tested, 2000 cells in 100 μL of medium were plated into each well of a 96-well plate. After an incubation period of 24 h after cell seeding, the polymers were added to the growth medium to achieve the target concentration in a range between 20 nM to 20 μM. The plates containing the cells were then incubated for 72 h at 37 °C in an atmosphere containing 5% CO_2_. Subsequently, 10 μL of a 0.25 mg mL^–1^ MTT solution in PBS (Invitrogen) was added to each well and blank absorbances were recorded using an Infinity 1000 plate reader (Tecan). Plates were incubated at 37 °C and 5% CO_2_ for 3 h to develop blue staining of the cells. The reaction was stopped by the addition of 150 μL of a 20% SDS solution and after 1 h of incubation at 37 °C, the cells and stain were dissolved to obtain a homogeneous solution. The final absorbance is read again using an Infinity 1000 plate reader (Tecan) and the relevant signal is counted as the difference from the blank absorbance. Data are normalized to the untreated control and plotted as data points with an error bar of the range using GraphPad Prism software.

#### 2.3.5 MTT Viability Assay with Lipid Nanoparticles

The MTT viability assay with lipid nanoparticles was performed with slight differences compared to the assay with polymers due to the short-term stability of the latter. For both cell lines (PC3 and MDA-MB-231), 100 μL of medium containing 20000 cells was plated into each well of a 96-well plate. After an incubation period of 24 h after cell seeding, 10 μL of a diluted LNP solution was added to the growth medium to achieve the target 5 μΜ concentration. The plates were incubated for 24 h at 37 °C in an atmosphere containing 5% CO_2_. Subsequently, 10 μL of a 0.25 mg mL^–1^ MTT solution in PBS was added to each well. The plates were incubated at 37 °C in an atmosphere containing 5% CO_2_ overnight to develop blue staining of the cells. The reaction was stopped by the addition of 150 μL of a 20% SDS solution and after 1 h of incubation at 37 °C, the cells and the stain were dissolved to form a homogeneous solution. The final absorbance was read again on the Infinity 1000 plate reader (Tecan). As previously, the relevant signal was counted as the difference from the blank absorbance. The data was normalized to the untreated control and plotted as mean ± standard deviation using GraphPad Prism software. Each experiment was carried out in triplicate. Statistical significance of the difference in viability was determined using a *t*-test against a hypothetical value of 100 with α = 0.05.

#### 2.3.6 Flow Cytometry Analysis of Cell-Polymer Interactions

To analyze cell-polymer interactions, cells from each cell line were cultured in a 48-well plate. To assess the specific interactions of polymers with glucose transporters, the cells were preincubated for 1 h with 200 μL of DMEM medium (Merck D5030) supplemented with 0.37 g L^–1^ sodium bicarbonate (Merck), with or without 27.6 mM glucose. Subsequently, 200 μL of a 200 nM polymer solution in glucose-free DMEM medium was added to each well, and the cells were incubated for an additional hour at 37 °C and 5% CO_2_. The cells were then harvested using a trypsin/EDTA solution and analyzed using a Novocyte Quanteon 4016 Combo (Agilent) cytometer. Live cells were gated, and the geometric mean of cy5 fluorescence was determined. Each measurement was performed in technical triplicate and biological duplicate. Individual biological replicates were plotted as geometric means of technical replicates and presented as mean values with standard deviation. The statistical significance of the potential difference between the incubation with and without glucose was assessed by performing a multiple *t*-test with Šidák-correction using the GraphPad Prism software.

#### 2.3.7 Flow Cytometry Analysis of Cell–Lipid Nanoparticle Interactions

To analyze cell–particle interactions, cells from each cell line were cultured in a 96-well plate. The cells were incubated in 100 μL DMEM medium with or without glucose. After 1 h, 100 μL of a 200 nM nanoparticle solution in glucose-free DMEM medium was added to each well and the cells were incubated for another hour at 37 °C in an atmosphere containing 5% CO_2_. The cells were then harvested using a trypsin/EDTA solution and analyzed using a NovoCyte Quanteon 4016 Combo (Agilent) cytometer. Live cells were gated, and the geometric mean of cy3 and cy5 fluorescence was determined. Each measurement was performed in technical and biological duplicate. Individual biological replicates were plotted as geometric means of technical replicates and presented with mean and standard deviation. The statistical significance of the potential difference between the incubation with and without glucose was assessed by performing a multiple *t*-test with Šidák-correction using the GraphPad Prism software.

#### 2.3.8 Confocal Laser Scanning Microscopy of Cell–Polymer and Cell–Nanoparticle Interactions

To analyze cell-polymer and cell–particle interactions, PC3 cells were cultured in glass-bottomed 4-chamber dishes (Cellvis, D35C4–20–1.5 N) at approximately 50% confluence. For assessing cell-polymer interactions, the cells were incubated for 1 h with 100 μL of a 50 nM polymer solution in serum-free DMEM media (Merck) supplemented with 4 mM glutamine at 37 °C in an atmosphere containing 5% CO_2_. To assess cell–nanoparticle interactions, the cells were incubated in 125 μL of glucose-free DMEM for 1 h at 37 °C and 5% CO_2_. Afterward, 125 μL of an 800 nM nanoparticle solution in glucose-free DMEM was added and incubated for another hour. The cells were then counterstained with 0.5 μg mL^–1^ Hoechst 34580 solution (Thermo Scientific) for 5 (polymers) or 10 (nanoparticles) minutes and washed with phosphate buffer solution. Fluorescence images were captured on a Stellaris 5 (Leica) confocal microscope and processed using ImageJ software.^74^

## 3 Results and Discussion

### 3.1 Synthesis of Glucosylated poly(2-Oxazolines)

**Scheme 1:**
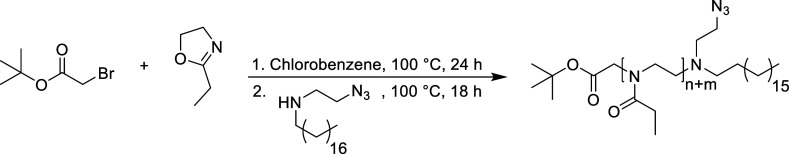
CROP of 2-Ethyl-2-oxazoline with *tert*-butyl bromoacetate as Initiator The polymerization was quenched with *N*-(2-azidoethyl)octadecan-1-amine to introduce both a hydrophobic and a reactive moiety at the chain end.

A series of poly(2-oxazoline)s with two orthogonal reactive sites, a varying amount of glucose side groups, and a hydrophobic anchor to enable micellization as well as stabilization of lipid nanoparticles in aqueous systems using the polymer as macromolecular surfactant was synthesized *via* cationic ring opening polymerization (CROP). A terminal carboxylic acid group, which can be activated and coupled to molecules containing an amine group, was obtained by using *tert*-butyl-2-bromoacetate as initiator for the polymers P1 and P2 in the CROP of 2-ethyl-2-oxazoline (Scheme 1).^47^ Polymerizations were carried out in chlorobenzene at 100 °C for 24 h in a glovebox under nitrogen. Subsequently, the secondary amine *N*-(2-azidoethyl)octadecan-1-amine (2 eq, referred to as C_18_ in the written abbreviation of the polymer structures) was added to terminate the polymerization. The synthesis of this termination agent introducing a hydrophobic moiety as well as a reactive azide group for further functionalization at the same terminus of the polymer is described in the experimental section. Displacement of the bromine end group, or the counterion in CROP, with the amine was carried out at 100 °C for 18 h. The resulting crude products were precipitated in cold diethyl ether and dialyzed (RC membrane, MWCO 1000 Da, ethanol/CHCl_3_ 1:1) to obtain the pure products P1 and P2 with a DP of 33 and 55, respectively (Figures S2 and S3 and Table S1). A narrow molecular weight distribution was obtained even though CROP in chlorobenzene and/or with bromide as a counterion can suffer from slow initiation. Nevertheless, satisfying results were obtained in our group using this method beforehand, as well as for the presented system.^26,47^

**Scheme 2:**
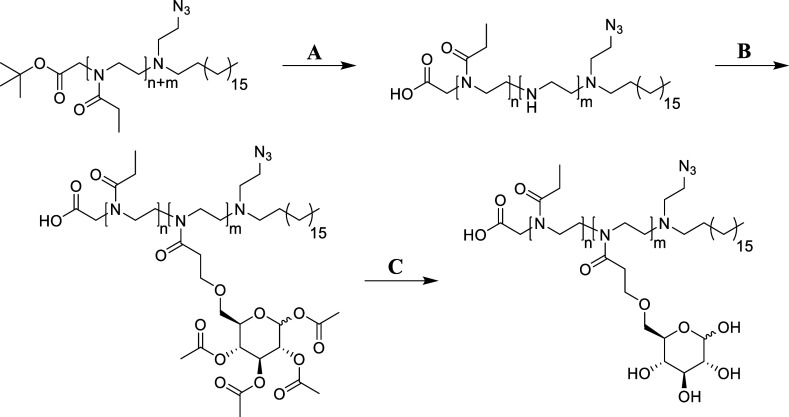
Partial Substitution of Ethyl Oxazoline Side Groups with Glucose Units A: 6 M HCl (aq), 100 °C, 20–360 min; B: tetraacetylglucose-6-COOH, PyBOP, DIPEA, DMF, rt, 24 h; C: NaOMe/MeOH, rt, 6 h, or 0.25 M NaOH (aq), rt, 1 h, then Amberlite IR120H^+^.

**Figure 1:**
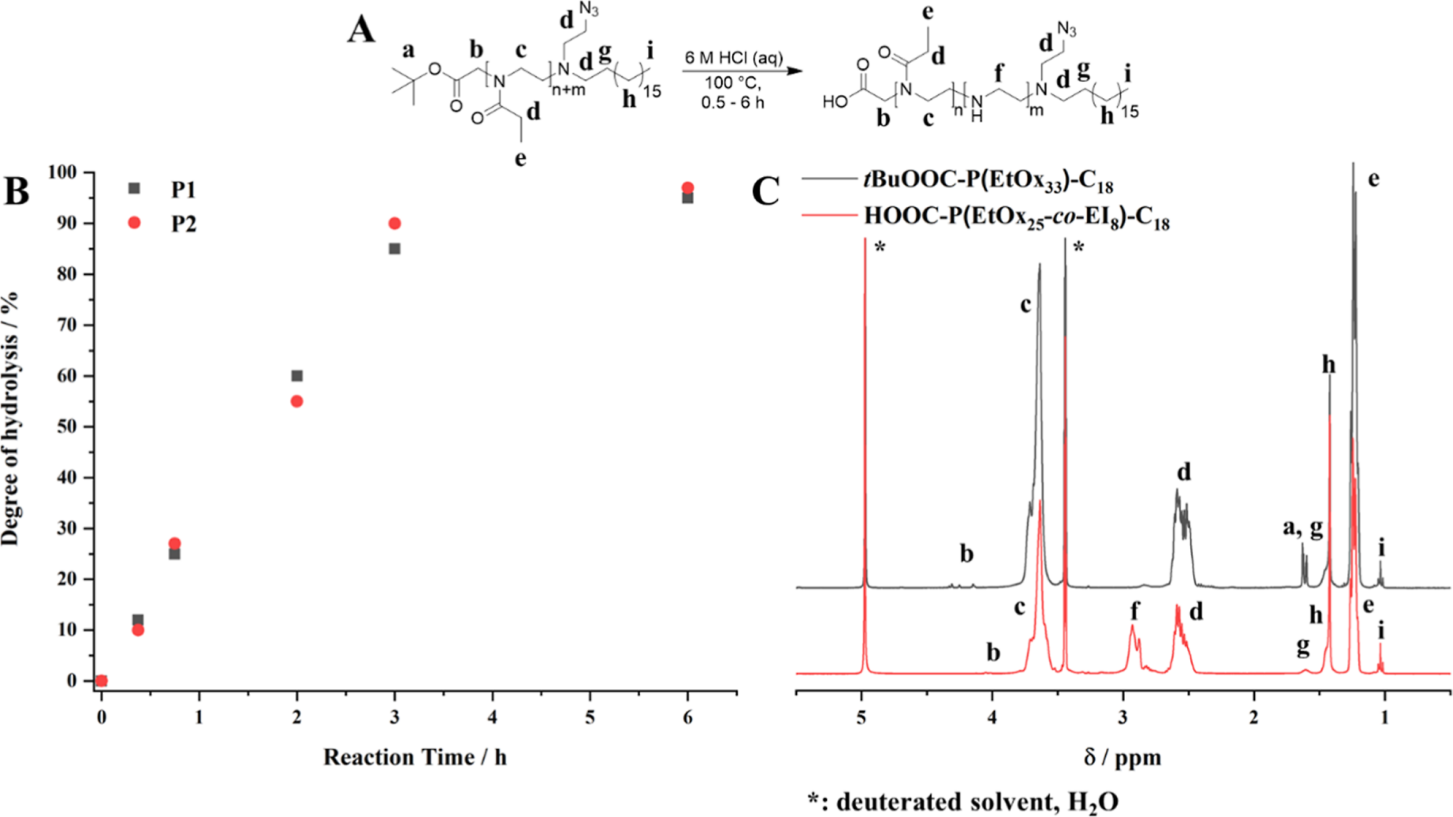
Partial acidic hydrolysis of polymers P1 and P2 leads to HOOC-P(EtOx_n–x_-*co*-EI_x_)_n_-C_18_ (A). The degree of hydrolysis was adjusted by varying the hydrolysis reaction time (B) and was determined by the ^1^H NMR signal ratio of protons neighboring amide units (c) or amine units (f) in the polymer backbone (C).

The glucose side groups were attached in a postpolymerization modification reaction including three steps (Scheme 2). In the first step, partial hydrolysis of the propionate side groups was carried out. For this purpose, the polymers were dissolved in 6 M aqueous hydrochloric acid, and heated to 100 °C for 20–360 min, depending on the desired degree of hydrolysis (Figure 1).^44^ Residual salts and propionic acid were removed during the purification process (neutralization, drying, resuspension in methanol and purification *via* Sephadex LH20 column in methanol). The *tert*-butyl ester was deprotected to release the free carboxylic acid during this step as well to obtain HOOC-P(EtOx-*co*-EI)-C_18_ (Scheme 2A, Table 1, Figures 1 and S4).

**Figure 2:**
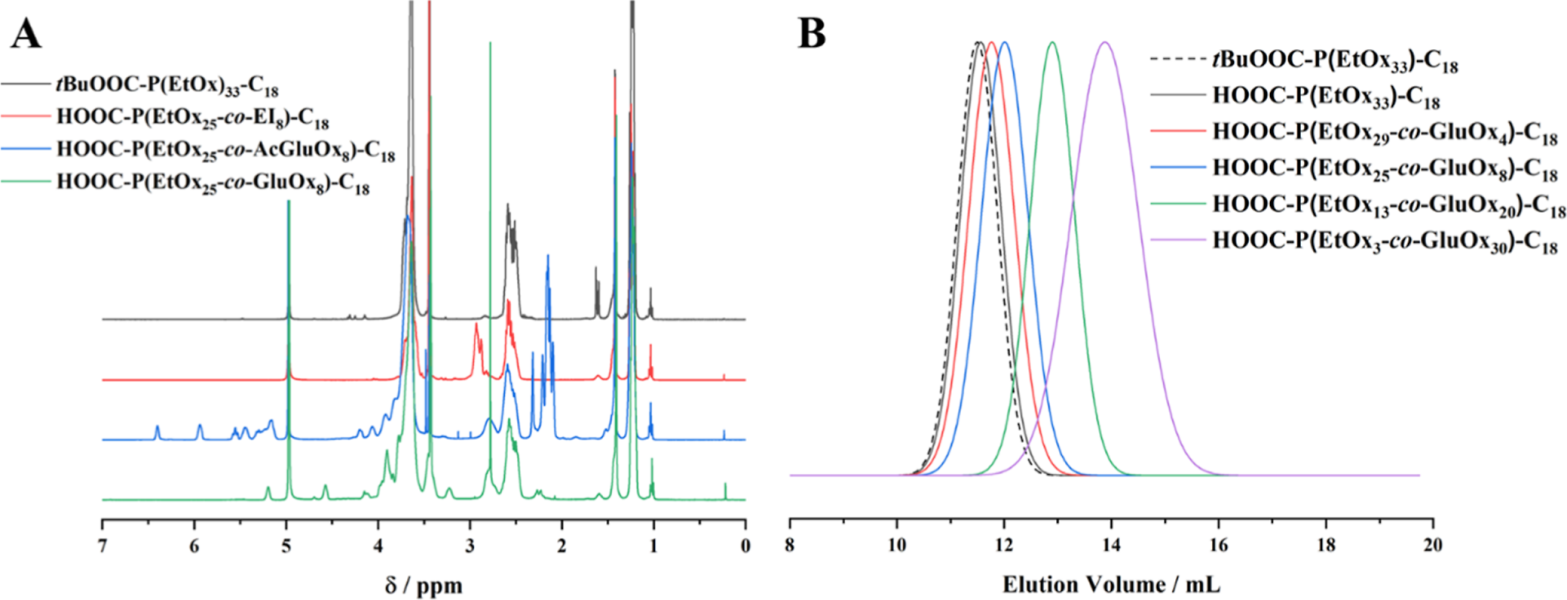
^1^H NMR spectra (here: MeOD) allowed to follow the synthesis of the glucosylated poly(2-oxazoline)s throughout all reaction steps (A, see Figures S3–S6 for peak assignment). GPC measurements of the final products in suitable solvents (here: products deriving from P1, MeOH/acetate buffer (pH = 6.0, 8:2)) show monomodal molecular weight distributions with narrow dispersities (B, further GPC traces can be found in Figure S7).

In the second step, 1,2,3,4-tetra-*O*-acetyl-d-glucose-6-propanoic acid was attached *via* an amide coupling reaction (Scheme 2B and Figure S5). As the hydroxyl groups at the C1, C3, and C4 site of glucose are crucial for interaction with and binding to GLUT1, glucose was connected to the polymer backbone in the C6-position. Stereoselective protection and functionalization of D-(+)-glucose was realized in 5 steps as described in the experimental section. A slight excess of the obtained glucose derivative compared to the free secondary amine groups/ethylene imine units was converted to an active ester using PyBOP in DMF and subsequently coupled to the secondary amine groups of the polymers to obtain HOOC-P(EtOx-*co*-AcGluOx)-C_18_.^45^ The products were purified on a Sephadex LH20 column before the acetyl protecting groups of the glucose side groups were removed in a last step using either sodium methoxide in methanol for products P1–1, P1–2, P2–1, and P2–2, or 0.25 M NaOH for all other polymers (Scheme 2C). The two different methods were chosen due to the ease of handling of the reaction and the subsequent purification process when carried out in methanol compared to the process in aqueous solution. Nevertheless, as only the aforementioned products were fully soluble in methanol, an alternative process was used for all other compounds which precipitated during the deprotection process with sodium methoxide in methanol. Depending on their solubility, the final products were purified on a Sephadex LH20 column in methanol or a Sephadex G25 column in micropure water, and subsequently freeze-dried from micropure water to obtain pure HOOC-P(EtOx-*co*-GluOx)-C_18_ of different DP and composition. ^1^H NMR spectroscopy allowed to follow the reaction during all steps and proved full replacement of the hydrolyzed propionate groups (Figures 2A, S5 and S6 and Table 2). GPC measurements in MeOH/acetate buffer (pH = 6.0, 8:2) showed monomodal molecular weight distributions and narrow dispersities for all polymers but P1–4, P1–5, and P2–5 (Figures 2B and S7A), which could not be analyzed using this eluent due to the low solubility of polymers with a high degree of functionalization with sugar moieties in methanol. The dispersities and the molecular weights given in Table 2 were determined using a multiangle light scattering (MALS) detector. Due to the decreasing solubility of the compounds in the chosen GPC solvent with increasing degree of functionalization, reasonable values for *M*_n_ and D̵ were obtained for P1–0 – P1–2 and P2–0 – P2–2 only. Therefore, additional GPC measurements were carried out using DMSO as a solvent. The measurements yielded reasonable dispersities and molecular weights for P1–3 – P1–5 and P2–3 – P2–5. The MALS peaks of P1–0 – P1–2 and P2–0 – P2–2 overlapped with the water peak in the chromatogram using DMSO as an eluent and were therefore not suitable for the determination of molecular weight and dispersity. In general, it is visible that the GPC peaks of the polymers in DMSO were close to the lower molecular weight limit of the system, which caused an ill-shaped or noisy trace toward higher elution volumes. The additional GPC traces of the polymers in DMSO are depicted in Figure S7B. Choosing a completely aqueous eluent system for GPC measurements was avoided due to the hydrophobic end group of the polymers. Partial aggregation or influence on the coiling behavior of the polymers to prevent contact in between the hydrophobic end group and the aqueous environment can be expected, which is not desired in GPC measurements.

**Table 2:** Composition, Molecular Weight and Dispersity of all Polymers of the Formula HOOC-P(EtOx_n–x_-*co*-GluOx_x_)_n_-C_18_, as Determined by ^1^H NMR Spectroscopy and GPC Measurements (MALS Detector)

	composition	ratio EtOx/GluOx	*M*_n_	*M*_n_	D̵	*M*_n_	D̵
		^1^H NMR^a^		GPC^b^		GPC^c^	
P1–0	HOOC-P(EtOx_33_)-C_18_	100:0	3700	3400	1.07	trace at lower *M*_n_ limit	
P1–1	HOOC-P(EtOx_29_-*co*-GluOx_4_)-C_18_	88:12	4400	4300	1.08	trace at lower *M*_n_ limit	
P1–2	HOOC-P(EtOx_25_-*co*-GluOx_8_)-C_18_	75:25	5100	4600	1.06	trace at lower *M*_n_ limit	
P1–3	HOOC-P(EtOx_13_-*co*-GluOx_20_)-C_18_	40:60	7200	compound not sufficiently soluble		4100	1.42
P1–4	HOOC-P(EtOx_3_-*co*-GluOx_30_)-C_18_	10:90	9000	compound not sufficiently soluble		5800	1.34
P1–5	HOOC-P(EtOx_1_*-co*-GluOx_32_)-C_18_	3:97	9400	compound not sufficiently soluble		10100	1.61
P2–0	HOOC-P(EtOx_55_)-C_18_	100:0	5800	6200	1.04	trace at lower *M*_n_ limit	
P2–1	HOOC-P(EtOx_49_-*co*-GluOx_6_)-C_18_	90:10	6900	7600	1.06	trace at lower *M*_n_ limit	
P2–2	HOOC-P(EtOx_40_-*co*-GluOx_15_)-C_18_	73:27	8500	9100	1.05	trace at lower *M*_n_ limit	
P2–3	HOOC-P(EtOx_25_-*co*-GluOx_30_)-C_18_	45:55	11200	compound not sufficiently soluble		6800	1.43
P2–4	HOOC-P(EtO*x*_6_-*co*-GluOx_49_)-C_18_	10:90	14600	compound not sufficiently soluble		11500	1.40
P2–5	HOOC-P(EtOx_1_-*co*-GluOx_54_)-C_18_	3:97	15500	compound not sufficiently soluble		42600	2.33

a : P1–0 – P1–2 and P2–0 – P2–2: CDCl_3_; P1–3 – P1–5 and P2–3 – P2–5: DMSO-*d*_6_.

b : GPC solvent: MeOH/acetate buffer (pH 6.0), 8:2; d*n*/d*c* = 0.18.

c : GPC solvent: DMSO; d*n*/d*c*: 0.15.

As P1 and P2 without d-glucose side groups were used for further experiments as well, the *tert*-butyl protecting group of the carboxylic acid was removed by stirring the polymer in a mixture of trifluoroacetic acid and water (95:5) for 1 h and subsequent purification of the products *via* a Sephadex LH20 column in methanol to exclude a possible influence of the hydrophobic end group compared to the free acid group in particle formation and biological experiments (Table 2).

### 3.2 Micellization and Stabilization of Solid Lipid Nanoparticles

All polymers presented in Table 2 were completely soluble in water. The pH values of the polymer solutions ranged from 7.2 to 7.8, demonstrating that the potentially ionizable groups do not influence the pH value of the solution significantly. The number-weighted hydrodynamic radius of their solution structures was determined *via* DLS measurements and ranged in between 2.3 and 5.9 nm (Table 3 and Figure S8). With the contour length of a 2-oxazoline monomer unit being ≈0.32 nm, this leads to the assumption that despite the hydrophobic end group of the polymers, no defined micelles with small hydrophobic cores were formed. Instead, single-molecule aggregates that are able to shield the hydrophobic anchor of the polymer from the aqueous environment were present in the solutions.^75^ This was surprising, as similar structures were reported to form micelles in aqueous solutions at significantly lower concentrations.^76−80^ Also, the shape of the DLS correlation functions indicates the additional presence of aggregates, especially for polymers with a lower degree of functionalization (Figure S9). These are probably loose aggregates with low stability, and the fact that they are not visible in the number-weighted DLS CONTIN plot suggests that their number can be neglected compared to the number of single-molecule aggregates. Nevertheless, this equilibrium may be shifted in salt solutions or biological fluids, which is relevant in a biomedical context. As the latter is the intended field of application of these polymers, the micellization behavior was further investigated in PBS and DMEM. As preliminary tests of the micellization behavior in PBS and DMEM showed similar results, all presented studies were carried out in DMEM at 37 °C to mimic the conditions of *in vitro* experiments. The formation of aggregates under *in vitro* conditions was visible in nile red encapsulation experiments. While pristine nile red solutions in DMEM only showed negligible fluorescence due to quenching in aqueous environments, nile red fluorescence increased with increasing concentration of polymer, indicating micellization. The highest intensities throughout the measurements were detected for polymers with a lower degree of functionalization with glucose, supporting the assumption that these species are more likely to aggregate due to their higher flexibility and lower hydrophilicity. For polymers with a higher degree of functionalization (P1–3 – P1–5 and P2–3 – P2–5), a sudden change in rate of the increase of the fluorescence intensity suggests that the critical micelle concentration was reached in between 0.25 and 0.5 mg mL^–1^ within the investigated concentration range (Figure S10A,B). At a concentration of 1 mg mL^–1^ in DMEM at 37 °C, all polymers formed micellar structures (Figure S10C,D).

**Table 3:** Hydrodynamic Radii of P1–0 – P1–5 and P2–0 – P2–5 without Additive or Used as Stabilizer for Tetradecan-1-ol/Cholesterol Nanoparticles in Aqueous Solution^a^

	<*R*_H_>_*n*, app_/nm, polymer in H_2_O	<*R*_H_>_*n*, app_/nm, LNP	stability
P1–0	2.8	11.8	3–4 d, 8 °C
P1–1	3.1	12.9	3–4 d, 8 °C
P1–2	2.3	17.2	3–4 d, 8 °C
P1–3	3.5	15.8	3–4 d, 8 °C
P1–4	3.5	17.2	3–4 d, 8 °C
P1–5	2.5	9.8	3–4 d, 8 °C
P2–0	3.8	13.6	3–4 d, 8 °C
P2–1	5.9	12.9	3–4 d, 8 °C
P2–2	3.6	17.0	3–4 d, 8 °C
P2–3	3.6	15.5	3 d, 8 °C
P2–4	4.4	21.5	1 d, 8 °C
P2–5	2.9	10.2	<1 d
control	no polymer	15.9	<1 h

a While aqueous solutions of the polymers were stable, solid lipid nanoparticles often decomposed over time, therefore their stability upon storage of the particle solution in the fridge is stated.

In biological applications, nonspecific binding, which can occur *via* hydrophobic moieties, must be avoided at least until the particle or functional polymer has reached its site of action. In addition to forming small micellar structures depending on the polymer concentration, the degree of functionalization and the surrounding medium, the presented polymers can act as surfactants to stabilize hydrophobic particles or droplets in aqueous solutions. In this manuscript, LNP were generated from tetradecan-1-ol and cholesterol and stabilized with the presented polymers. The stability of these nanoparticles did not only depend on polymer properties (e.g., on the ratio of hydrophobic to hydrophilic moieties in one chain, or its coiling behavior), but also on the lipid chosen as nanoparticle core. Tetradecan-1-ol was chosen as the main component of the lipid mixture as its melting temperature is 38 °C. Therefore, the particles were prepared from the polymer and a tetradecan-1-ol melt, but it is assumed that they exhibit solid cores under physiological conditions. Slightly increased temperatures increase the flexibility of the stabilizers on the particle surface and allow for stronger interactions with the biological environment. Further, tetradecan-1-ol is biocompatible and degradable under physiological conditions after metabolization to the corresponding fatty acid (i.e., tetradecanoic - myristic acid).^81^ Cholesterol as a naturally abundant molecule in the human body is biocompatible as well and prevents crystallization of tetradecan-1-ol, which presumably led to displacement of the surfactant from the lipid core during preliminary experiments.

**Figure 3:**
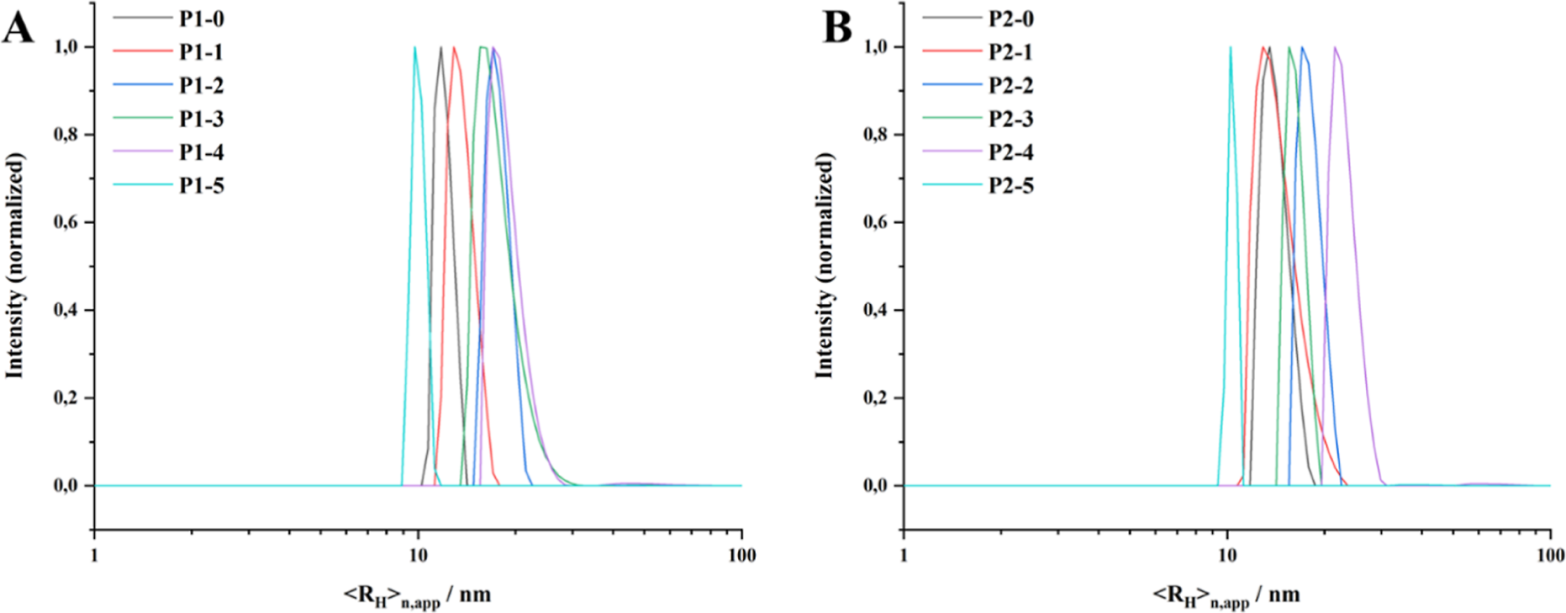
Number-weighted DLS CONTIN plots of LNP generated from tetradecan-1-ol and cholesterol (3:1) and polymers P1–0 *–* P1–5 (A) and P2–0 *–* P2–5 (B). Intensity-weighted DLS CONTIN plots for these measurements are depicted in Figure S11.

**Figure 4:**
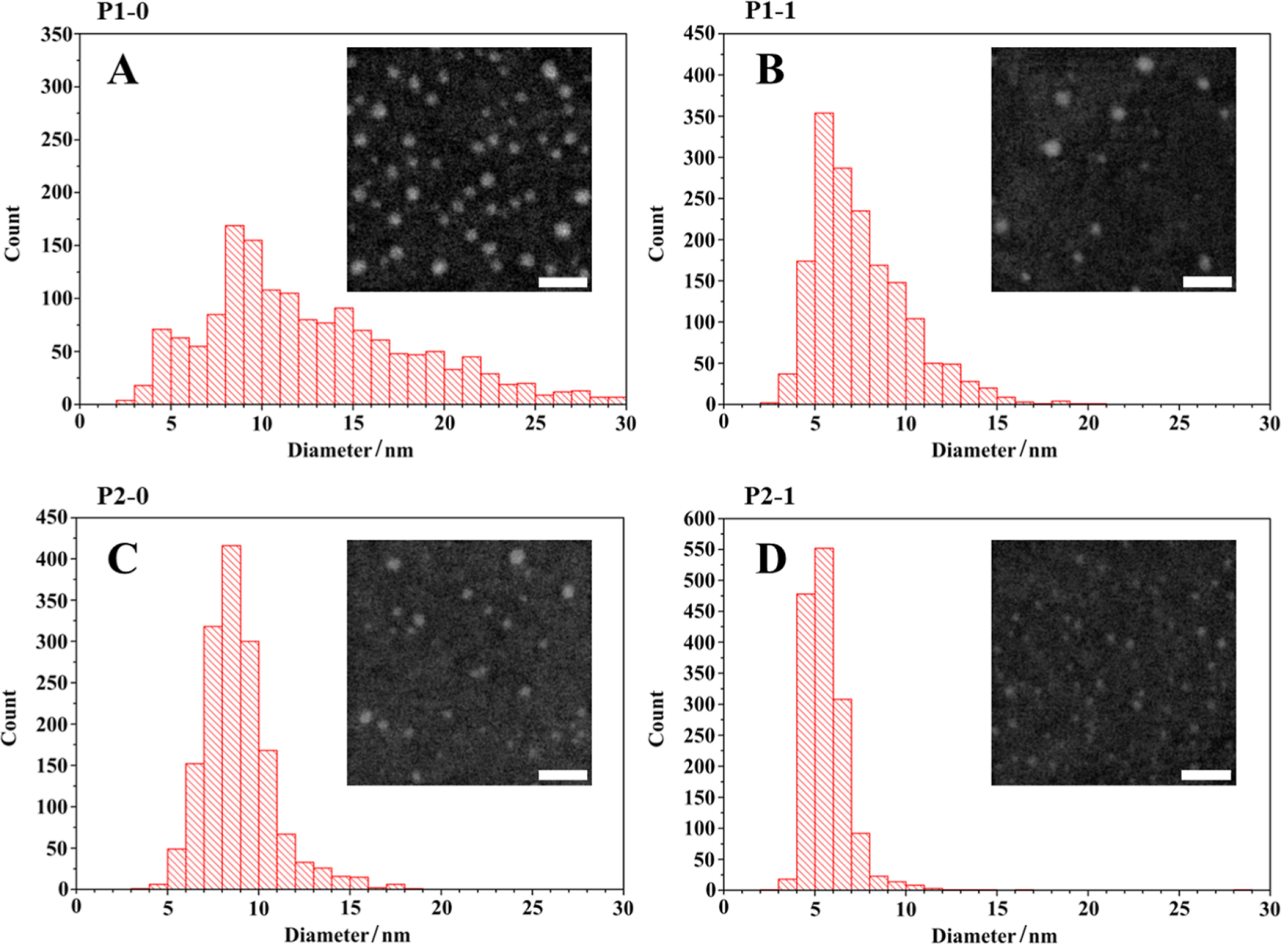
Particle (core) size distribution for LNP generated from P1–0 (A), P1–1 (B), P2–0 (C), and P2–1 (D). Inlay: STEM micrographs for the respective particles, scale bar 50 nm.

The particles were generated by dissolution of the respective polymer in water at 50 °C, and addition of the solution to a melt of tetradecan-1-ol with cholesterol dissolved in it (3:1, 50 °C). The mixture was stirred and subsequently ultrasonicated. Number-weighted DLS CONTIN plots of the obtained particles, with hydrodynamic radii ranging from 10–20 nm, are depicted in Figure 3. Exemplary STEM measurements revealed an average diameter of the particle core of 5–10 nm (Figure 4). The particle radius obtained from STEM measurements was significantly smaller than the one obtained from DLS measurements, as STEM micrographs depict only the densely packed lipid core of the particle.

**Figure 5:**
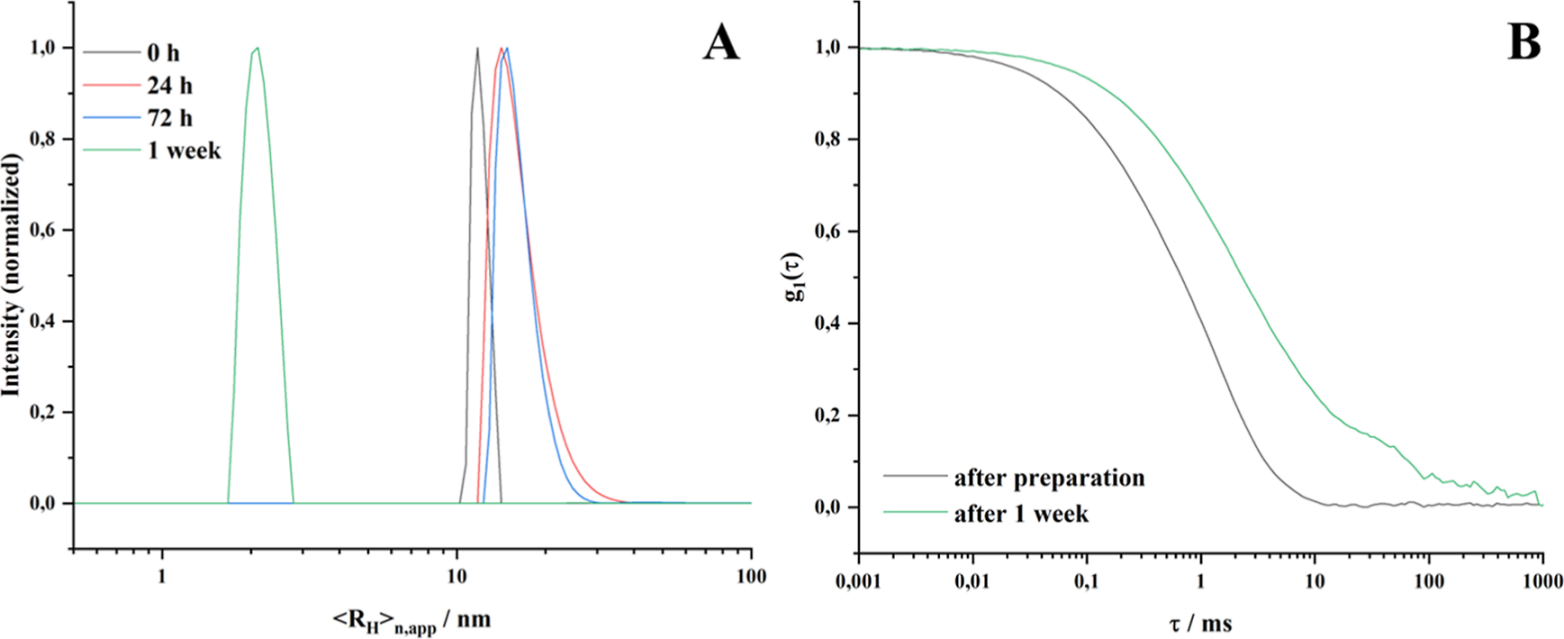
Number-weighted DLS CONTIN plots of LNP prepared from P1–1 at different time points after preparation. The majority of the particles decomposed after 1 week, which is indicated by a shift of < *R*_H_ > _*n*,app_ to lower values as well as a characteristic change in the correlation function g^1^(τ). DLS CONTIN plots for all other LNPs are depicted in Figures S12 and S13.

LNP generated from the mixture of cholesterol and tetradecan-1-ol without stabilizer exhibited a similar hydrodynamic radius, yet these particles aggregated rapidly (within 1 h after preparation) both at room temperature as well as in the fridge at 4 or 8 °C. Stabilization of LNP with the glycopolymers extended their shelf life to an average of 3–4 days at 8 °C. After that, displacement of the stabilizer from the solid lipid particle core led to aggregation and precipitation of the lipid, which can be demonstrated *via* DLS measurements at different time points after particle preparation: The number-weighted hydrodynamic radius decreases upon displacement and self-organization of the polymer in aqueous solution (Figures 5, S12 and S13). In general, particles stabilized by glycopolymers based on P2 exhibited a lower shelf life, which can be attributed to the fact that the hydrophobic anchor of the longer polymer chains may be less accessible. Additionally, the shelf life of the particles decreases for polymers with a high glucose content, which may be attributed to two factors: The higher rigidity of the hydrophilic polymer chain due to an increased steric demand of the glucose side groups in comparison to the propionate side groups, and the increased hydration of the polymer chain with increasing glucose content, caused by the four free hydroxyl groups per monomer.^43,82^ Those factors could prevent beneficial arrangement of the polymer chain on the surface of the nanoparticle.

To investigate the stability of the particles under the conditions of a biological experiment, short-time stability studies in DMEM at 37 °C were carried out. The hydrodynamic radius of the particles in DMEM appeared to be slightly larger than in water, which may be attributed to the attachment of small molecules to the particle surface. The zeta potential of the particles was slightly negative (0 – −5 mV), as it would be expected due to the negatively charged carboxylic acid end groups of the polymeric stabilizer that were exposed on the particle surface to the surrounding medium. It was visible that the number weighted and the intensity weighted hydrodynamic radius as well as the zeta potential of the particles was stable for at least the time of the biological studies carried out in this manuscript (Figures S14 and S15). Differences in the stability of the different LNP were detected in DLS, yet they did not correlate with the molecular weight or the degree of glucosylation of the samples (Table S2).

### 3.3 Fluorescent Labeling of Polymers and SLNP

**Figure 6:**
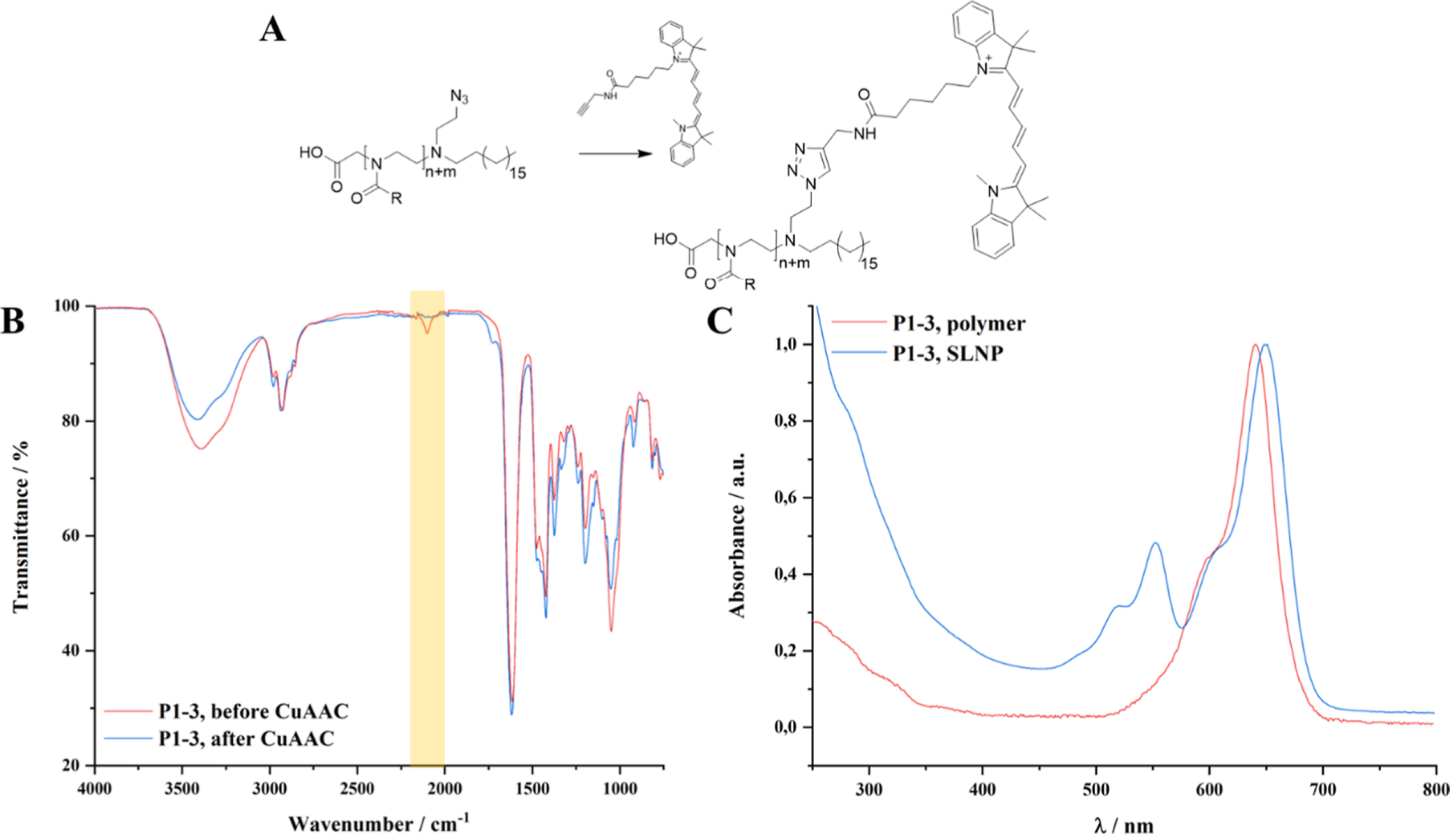
Reaction scheme of the attachment of cy5 to the functional poly(2-oxazoline) *via* CuAAC (conditions: CuSO_4_ × 5H_2_O, sodium ascorbate, DMSO, 24 h, rt; A). FTIR spectra show conversion of the azide group (yellow region, B), and UV/vis spectra in aqueous solution depict the typical cy5 spectrum (C, an overlay with an UV/vis spectrum of the corresponding SLNP containing 10% cholesterol-cy3/cholesterol is shown), as exemplified for polymer P1–3.

In the next step, interaction with and uptake of the compounds into cells, and the influence of the DP and glucosylation on these processes, was investigated. To be able to track the polymers in biological studies, a cyanine 5 (cy5) dye with alkyne functionality was connected to the azide group adjacent to the hydrophobic anchor. The dye was synthesized from cy5 carboxylic acid, prepared as described in an earlier publication, *via* coupling with propargyl amine.^60^ Subsequently, the functional dye was reacted with the polymer using copper-catalyzed azide–alkyne coupling (CuAAC). The consumption of the azide group during CuAAC was proven by FTIR (Figures 6 and S16).^83^ The characteristic azide stretching band at 2100 cm^–1^, which was visible in the FTIR spectra of the nonlabeled polymers, disappeared upon attachment of cy5. The absence of free dye was proven by GPC measurements (MeOH/acetate buffer (pH = 6.0), 8:2) using a UV detector (λ = 650 nm). Only the sample P1–0 showed slight contamination with free dye. Due to the low solubility of the polymers with a high degree of functionalization in the solvent used, the respective GPC traces were only used for the purpose of proving the absence of free dye (Figure S17). The degree of functionalization was determined *via* UV/vis-measurements (Figures 6, S18 and Table S3).

Additionally, cholesterol was partially replaced with a cyanine 3 (cy3) labeled cholesterol derivative to track particle integrity during biological experiments. The ideal amount of cy3-labeled cholesterol was determined to be 30%, as lower amounts of cy3 present in the samples were not detectable due to fluorescence resonance energy transfer (FRET) interactions between cy3 and cy5 in the densely packed lipid core.^84^

Functionalization of the polymeric stabilizer with a fluorescent dye further allowed to verify the analytical results on the particle size populations in the LNP samples and aqueous polymer solutions obtained from DLS measurements *via* fluorescence correlation spectroscopy (FCS). DLS measurements showed that the LNP samples exhibit a larger hydrodynamic radius than the samples containing the pristine polymer, as the LNPs are larger than the micelles formed from the polymers without encapsulation of the lipid, or the free polymer. Nevertheless, it cannot be excluded that LNP samples contain polymeric micelles as a second species. Exemplary FCS measurements were carried out for polymer P2–2, and LNPs stabilized with this polymer. The obtained correlation functions were fitted as one-component diffusion with a triplet relaxation of the cy5 dye. The fit yielded diffusion coefficients and corresponding hydrodynamic radii that confirm the DLS results (*D*_T,LNP_ = 24.2 μm^2^ s^–1^; *R*_H,LNP_ = 10.1 nm; *D*_T,polymer_ = 73.7 μm^2^ s^–1^; *R*_H,polymer_ = 3.3 nm). If a significant amount of a particle species with a different *D*_T_ would be present in one of the samples (e.g., free dye in the polymer sample, or free dye or free polymer in the LNP sample), notable differences in the obtained hydrodynamic radius would be expected.Therefore, it can be assumed that the LNP samples do not contain significant amounts of polymeric micelles or free polymer.

### 3.4 *In Vitro* Cell Assays

Both cell–polymer and cell–LNP interactions were investigated in *in vitro* cell experiments to assess the behavior of the synthesized compounds toward different cell lines. MDCK, PC3, and MDA-MB-231 cell lines were selected due to their GLUT1 expression reported in literature and our own assessment of the latter using an anti-GLUT1 antibody and analyzing its binding *via* flow cytometry (data not shown).^85−87^

**Figure 7:**
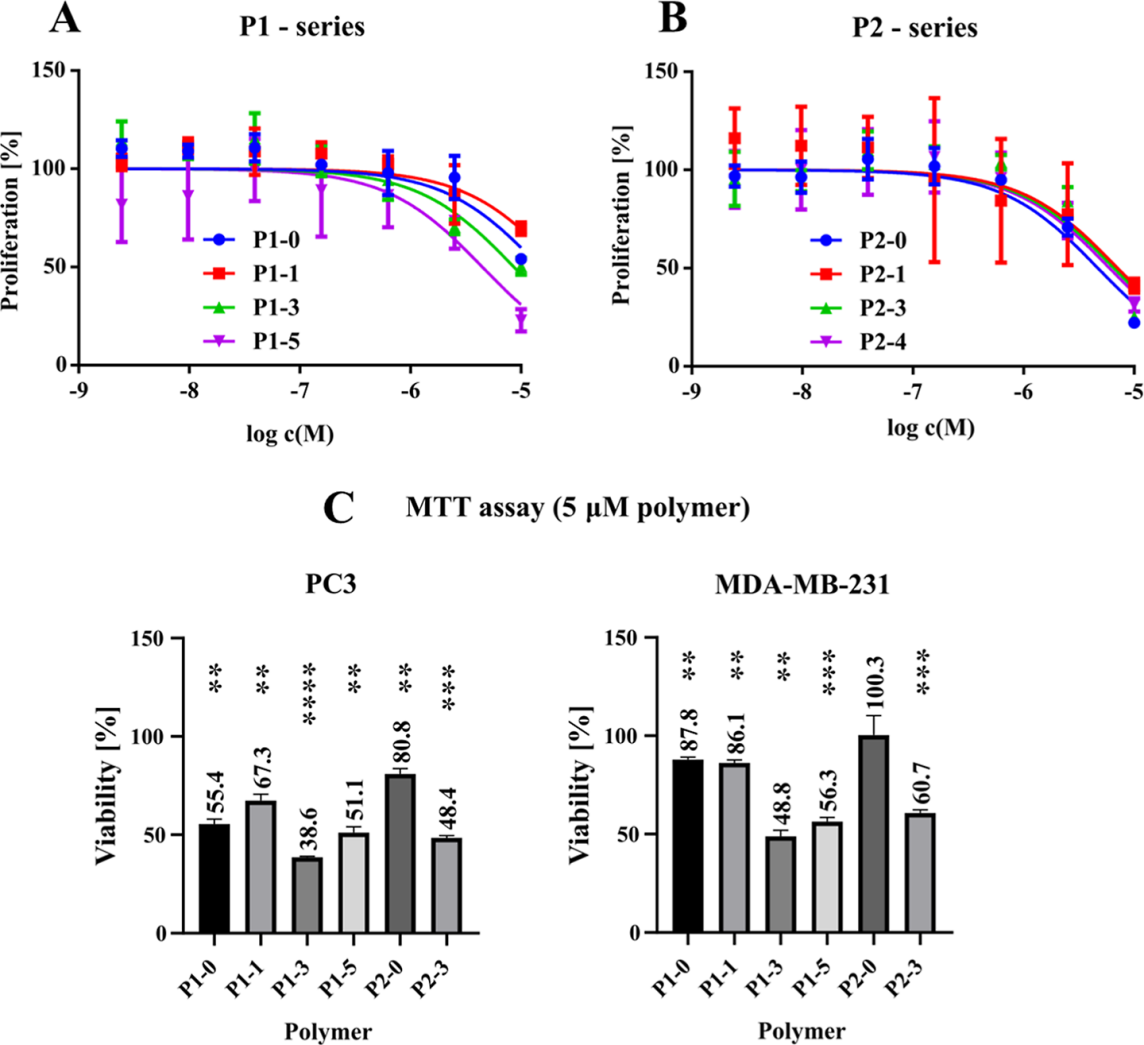
Proliferation and viability assays with polymers. Proliferation of MDCK cells as a function of polymer concentration, normalized to the untreated control, indicating decreasing cell proliferation with increasing amount of glucose in the polymers (A,B). Error bars represent ±SD (*n* ≥ 3) and the curve was fitted as log(concentration) vs normalized response. The MTT assay using MDA-MB-231 and PC3 cells confirmed these findings (C,D). Error bars represent ±SD (*n* = 3). MTT absorbance values ranged between 0.57 and 1.95 for PC3 cells, and 0.23 and 1.03 for MDA-MB-231 cells. Asterisks indicate the statistical significance of the difference from the hypothetical 100% value (**p < 0.01, ***p < 0.001, ****p < 0.0001).

In first experiments, the viability and proliferation of cells in the presence of cy5-labeled polymers was investigated. The results of the proliferation assay using MDCK cells to assess the toxicity of the polymers are shown in Figure 7A,B. Cells were incubated for 48 h with different concentrations of polymers. The polymers showed no toxic behavior at low concentrations (below 250 nM), while at micromolar concentrations they clearly inhibited proliferation. The actual inhibition of proliferation showed a dependence on the degree of glucosylation of the polymers. Polymers with a higher degree of glucosylation tended to inhibit MDCK cell proliferation more strongly in the P1 series.

To further investigate this phenomenon, we performed a more metabolically sensitive MTT assay with selected polymers on the PC3 and MDA-MB-231 cell lines. A polymer concentration of 5 μM was chosen as a concentration close to the IC50 values. The cells were incubated with the polymers for 72 h, the number of viable cells was determined and normalized to the untreated control. The assay confirmed decreased viability when cells were incubated with polymers containing higher amounts of glucose (Figure 7C,D).

The proliferation and viability tests indicate an acceptable *in vitro* toxicity of the compounds, even at concentrations leading to significant accumulation. Since all polymers have an antiproliferative effect at higher concentrations that is clearly dependent on the glucose content of the polymers, the toxic effects of the polymers could be explained by two independent mechanisms. First, the accumulation, or intercalation, of polymers at or into the cell membranes may decrease their metabolism and proliferative activity. Second, the uptake of glucose into the cells could be disturbed or inhibited by the presence of polymers with glucose units due to interactions of polymer-bound glucose molecules and glucose receptors.

**Figure 8:**
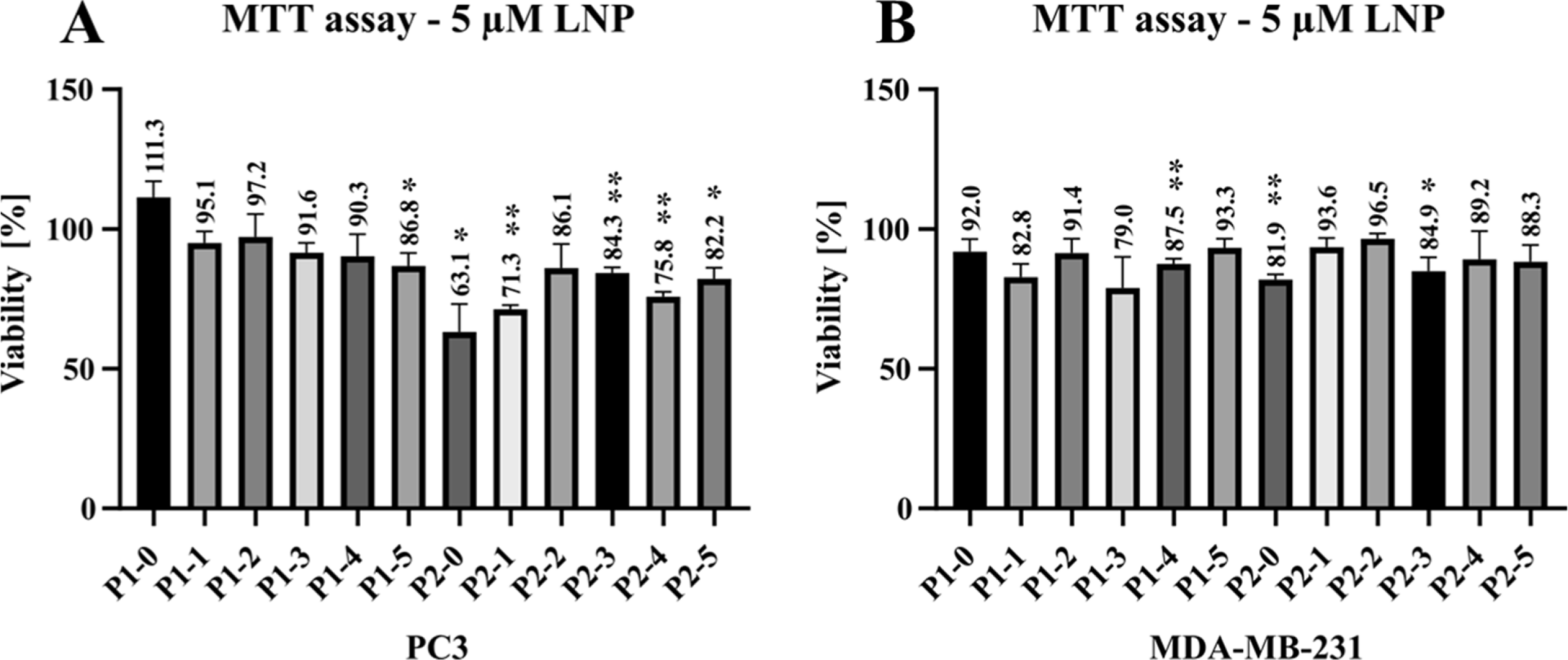
MTT viability assay after incubation of PC3 (A) and MDA-MB-231 cells with LNP. The data was normalized to untreated control and averaged. Error bars represent ±SD (*n* = 3). MTT absorbance values ranged between 0.25 and 0.4 for PC3 cells and 0.14 to 0.28 for MDA-MB-231 cells. Asterisks indicate the statistical significance of the difference from the hypothetical 100% value (*p < 0.05, **p < 0.01).

To also address the effects of LNP, in which the hydrophobic compartments of the polymers are masked, on cell metabolism, an MTT assay was performed. PC3 and MDA-MB-231 cells were incubated with LNP containing 5 μM of polymer for 24 h. The results are shown in Figure 8. Due to the shorter incubation time that was chosen due to the limited stability of the particles (24 h vs 72 h), the decrease in cell viability is less pronounced than in the case of the polymers. Still, some LNP stabilized with polymers of high glucose content derived from both P1 and P2 showed higher toxicity in PC3 cells (e.g., P1–5), but the effect was less pronounced than for the pristine polymers. The reason may be the same as for the decreased metabolic activity found for polymers with high glucose content–while the hydrophobic compartment is masked, glucose units may still interact with glucose receptors on the cell surface. The higher toxicity of nonglucosylated P2–0 on both cell lines was also surprising.

**Figure 9:**
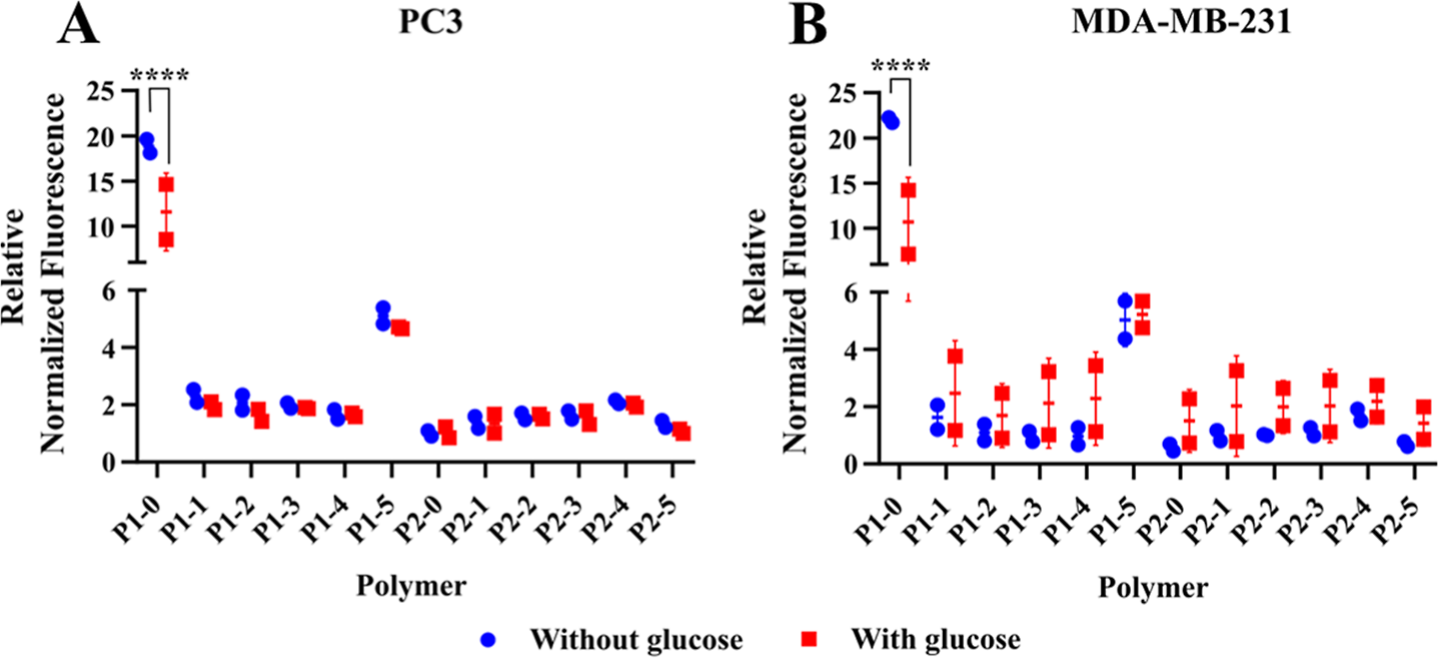
Results of cytometric analysis of the interaction of the polymers with PC3 (A) and MDA-MB-231 cells (B). Data were normalized to the untreated control and to the degree functionalization with cy5. Most polymers show a similar level of interaction with cells, except for the surprisingly high interaction of P1–0 and P1–5. Error bars represent ±SD (*n* = 2). The asterisks indicate the statistical significance of the difference between the signal obtained in DMEM with and without glucose (****p < 0.0001).

In the next step, cell–polymer interactions were investigated. PC3 and MDA-MB-231 cells were incubated with cy5-labeled polymers (100 nM final concentration) in DMEM with (13.8 mM) or without glucose. The harvested cells were then analyzed by flow cytometry for the presence of cy5 fluorescence. As each polymer has a different cy5 content, the UV/vis absorption of the samples was measured directly before the experiments and the respective results were normalized to the degree of functionalization with cy5 (Figure S19A). The highest nontoxic polymer concentration (100 nM) was used for the analysis of polymer–cell interactions. We did not observe any differences between uptake in glucose-free and glucose-containing media, except for a P1–0, which showed, to our surprise, a significantly lowered interaction with cells in glucose-containing media. P1–0, which is nonglucosylated, also showed the highest interaction with cells in general, compared to all other polymers that were studied. This hints toward the fact that polymer–cell interactions are strongly governed by the interactions of the hydrophobic compartments of the polymers (the hydrophobic anchor and also the neighboring cy5 dye).^60,88^ The combination of the lower DP of the P1 series and the lack of glucosylation may therefore lead to a high interaction of the hydrophobic anchor of the polymer with the hydrophobic compartments of the cell (e.g., cell membrane) without any competing effects. All other polymers showed a very similar level of interaction with cells. The surprisingly high signal of P1–5 could be attributed to the low degree of functionalization. The normalized results of the flow cytometry studies are depicted in Figure 9. The non-normalized data are shown in Figure S20.

To evaluate the possibility of reducing the nonspecific interaction of the lipid anchor of polymers with cells, we also analyzed the interaction of solid lipid nanoparticles (labeled by cholesterol-cy3) stabilized with the polymers. The particles were prepared as described above and contained both cy5 through the labeled polymers that were used, and cy3 attached to cholesterol as a label for the lipid core. Cells were incubated with the particles at a concentration of 100 nM with respect to the polymeric stabilizer in DMEM with (13.8 mM) or without glucose for 1 h. Harvested cells were then analyzed by flow cytometry. The results were again normalized to the level of cy3 and cy5 functionalization that was determined *via* UV/vis spectrometry prior to the experiments (Figure S19B,C).

**Figure 10:**
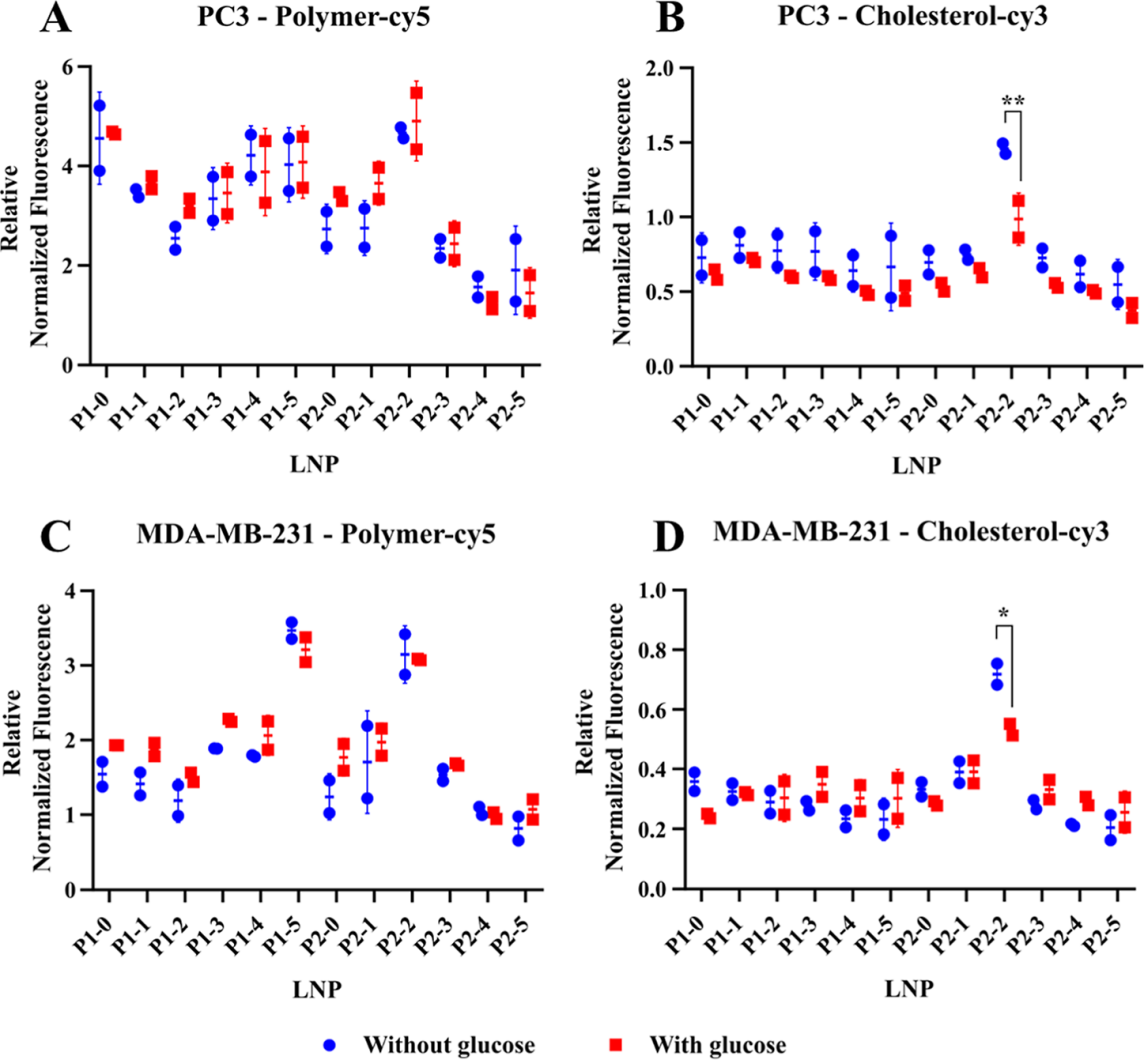
Results of cytometric analysis of the interaction of LNP (polymers labeled with cy5, A and C, and cholesterol labeled with cy3, B and D) with PC3 (A,B) and MDA-MB-231 cells (C,D). The results were normalized to the untreated control and to the degree of functionalization of the LNP with either cy3 or cy5. Most LNP show a similar level of interaction with cells, except for particles stabilized with P2–2, which also show a statistically significant different accumulation in the presence of DMEM media with and without glucose. Error bars represent ±SD (*n* = 2). The asterisks indicate the statistical significance of the difference between the signal obtained in DMEM with and without glucose (*p < 0.05, **p < 0.01).

Compared to polymers, solid lipid nanoparticles show less differences in particle–cell interactions due to nonspecific hydrophobic effects, as it was observed for P1–0 in flow cytometry experiments of the pristine polymers. In addition, the specific sample P2–2 showed comparably high particle–cell interactions, and showed increased accumulation based on the signal of cholesterol-cy3 when cells were incubated in glucose-free media. This suggests that the formulation of the polymers as LNP leads to at least partial masking of the lipid anchor, which may allow for glucose-mediated targeting. The results of the cytometric analysis are depicted in Figure 10, while the non-normalized data is shown in Figure S21.

**Figure 11:**
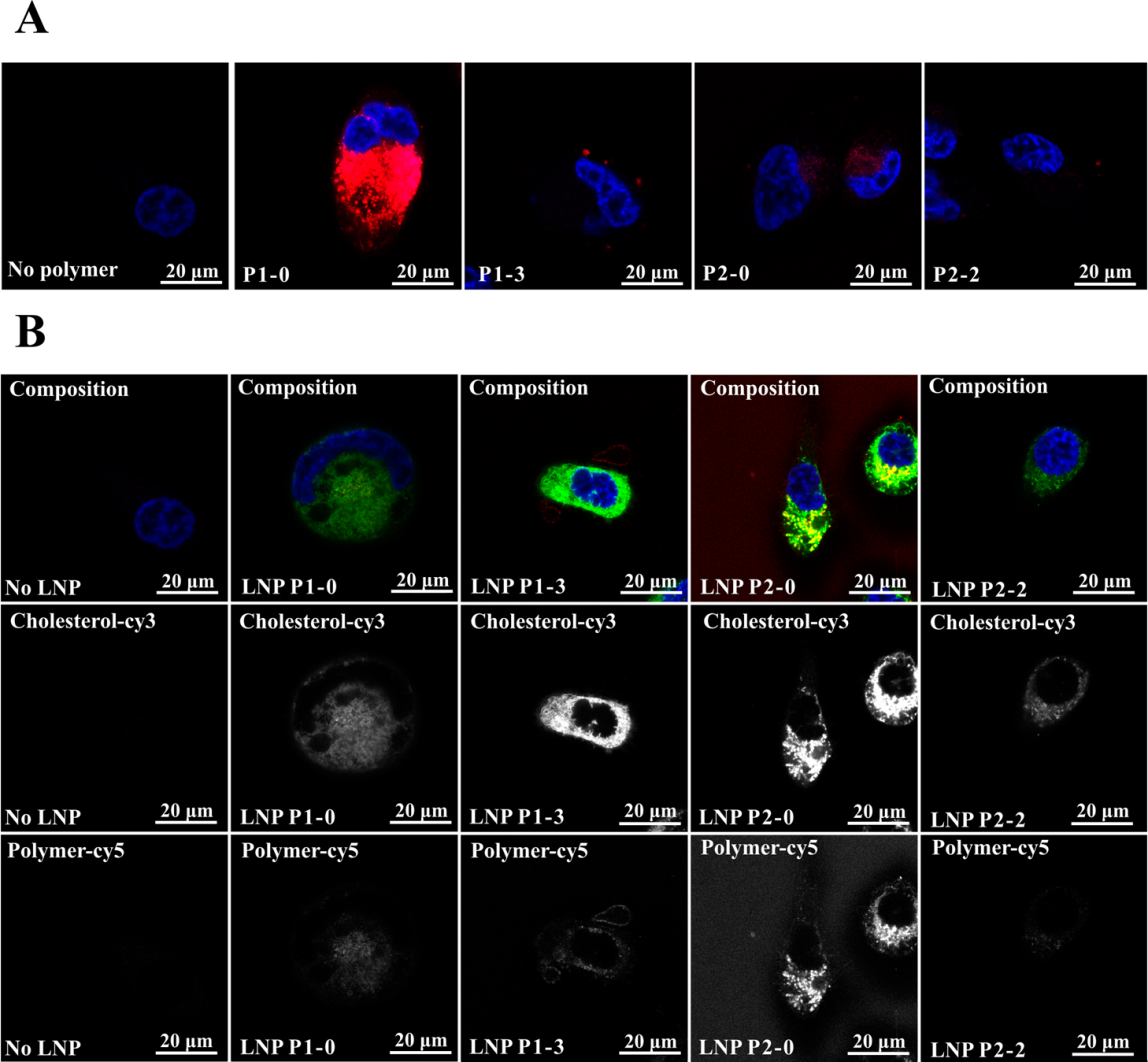
Confocal laser scanning microscopy of cell-polymer (A) and cell–nanoparticle interactions (B). (A): Blue channel: Hoechst 34580, laser wavelength 405 nm; red channel: cy5, laser wavelength 640 nm. (B): Blue channel: Hoechst 34580, laser wavelength 405 nm; Green channel: cholesterol-cy3, laser wavelength 561 nm; Red channel: polymer-cy5, laser wavelength 640 nm. For clarity, cy3-cholesterol and cy5-polymer monochrome images are shown below the composite images.

To further evaluate the biological interaction of polymers with cells at a subcellular level, we performed confocal laser scanning microscopy with selected polymers. PC3 cells were incubated with polymers at a final concentration of 400 nM in serum and glucose-free DMEM media and counterstained with Hoechst 34580. The acquired images of cells after incubation with selected polymers are shown in Figure 11A. Similar to the flow cytometry results, incubation with P1–0 resulted in a strong staining of the internal compartments of the cells. Polymers containing glucose (P1–3 and P2–2 in this case) also showed a greater tendency to produce larger spots, which may hint toward the uptake into lysosomes, while P1–0 and P2–0 distributed more homogeneously inside of the cells and attached to the hydrophobic compartments of the cell.

A similar approach was used to study the interaction with LNP as shown in Figure 11B. To investigate whether all components of the LNP interact in the same way, double-stained particles containing cy5-labeled polymer and cy3-modified cholesterol, prepared as described before, were used. PC3 cells were incubated with solid lipid nanoparticles at a polymer concentration of 400 nM in serum- and glucose-free DMEM and counterstained with Hoechst 34580. In accordance with the results obtained CLSM of the pristine polymer samples, cell-LNP interactions and uptake of the particles stabilized with P1–0 seemed to be lower than for the glucosylated counterpart P1–3. Further, the distribution of the fluorescence signal within the cells was more homogeneous. No significant difference in the distribution of the cy5- and the cy3-signal throughout the cells was observable. The only exception was detected in the sample containing LNP stabilized with P1–3, where increased cy5 fluorescence was detected in the cell membrane, which may hint toward the instability of the sample during the experiment.

In summary, it can be stated that the interaction of polymers with cells is not only based on specific interactions of glucose with GLUT1 receptors, but largely also on the physicochemical properties of polymers, mainly the interaction of the lipid anchor and the cy5 dye with the hydrophobic compartments of the cells (membranous compartments). Possibly, the hydrophobic properties of the lipidic anchor and the cy5 dye could lead to the formation of semistable aggregates, which do not only result in fluorophore quenching, but also in different cell uptake behavior. Both mechanisms lead to reduced reproducibility between measurements, especially with more hydrophobic polymers (polymers containing no or low amounts of glucose). On the other hand, formulation of the polymers as LNP can increase the impact of their hydrophilic, glucosylated compartment on polymer–cell interactions and uptake.

## 4 Conclusion

In this study, we synthesized multifunctional poly(2-ethyl-2-oxazoline)s containing two reactive end groups as well as a C_18_-based hydrophobic anchor on one end. Glucose functionalities were introduced by partial hydrolysis of the propionamide side groups and subsequent functionalization of the secondary amines with a reactive glucose derivative. Attachment of the glucose molecules through their C6-position was supposed to enable binding of the polymers to GLUT1 receptors. The polymers were used to stabilize LNP formulated from tetradecan-1-ol and cholesterol with their hydrophobic anchor. While the pure polymers showed no dependency of polymer–cell interactions on the degree of functionalization with glucose in flow cytometry experiments, as interactions rather depended on the physicochemical properties of these polymers, flow cytometry studies of LNP-cell interactions hinted toward an influence of the degree of functionalization with glucose on particle uptake. CLSM images demonstrated a rather homogeneous distribution of both polymers and LNP within the cells after uptake, which hints toward sufficient stability of the system for cell uptake in general. For the design of specific receptor targeting systems using the presented polymers, it is therefore also necessary to further minimize nonspecific interactions. Also, the excessive accumulation of the poly(2-oxazoline)s with their lipid anchor on cell membranes could be toxic at higher concentrations. We showed that despite the need to improve the stability of the LNP system, the presented glucose-decorated LNP may be promising drug transporters targeting GLUT1-expressing cells.

## Supporting Information Available

^1^H NMR spectra of target small molecule compounds; ^1^H NMR and MALDI-TOF spectra of all polymers after partial hydrolysis; ^1^H NMR spectra of the attachment of the protected glucose derivative, and after deprotection of the hydroxyl groups of glucose; GPC traces of target polymers based on P2 in MeOH/acetate buffer, GPC traces of all polymers that were not sufficiently soluble in MeOH/acetate buffer in DMSO; number weighted DLS CONTIN plots and DLS correlation functions of pure polymers in aqueous solution; investigation of micellization behavior of pristine polymers in DMEM at 37 °C; intensity-weighted DLS CONTIN plots of pure polymers and LNP in water; number-weighted DLS CONTIN plots of LNP in water at different time points after preparation; time-dependent zeta potential of the LNP in DMEM; time-dependent stability of LNP in DMEM; degree of functionalization of the polymers with cy5 based on UV/vis absorption; FTIR spectra of the polymers before and after the attachment of cy5-alkyne to the azide group *via* CuAAC; UV GPC traces of the polymer samples used in biological experiments; UV/vis spectra of the polymers used in biological experiments in aqueous solution; degree of functionalization of the samples used for biological studies with either cy5 or cy3; non-normalized flow cytometry results (PDF)
