# Supplementary material for: Solid Lipid Nanoparticles Coated with Glucosylated poly(2-oxazoline)s: A Supramolecular Toolbox Approach
Source: Biomacromolecules. 2025 Jan 8;26(2):861–82. doi: 10.1021/acs.biomac.4c01052 (PMC11815865; doi:10.1021/acs.biomac.4c01052)
Supplement: Supplementary file 1 — bm4c01052_si_001.pdf [file bm4c01052_si_001.pdf]

Supporting Information

Solid lipid nanoparticles coated with glucosylated poly(2-oxazoline)s: A supramolecular toolbox approach

Johanna K. Elter[a,*], *František Sedlák*[b], *Tomáš Palušák*[b], *Nicol Bernardová*[b], *Volodymyr Lobaz*[a], *Eva Tihlaříková*[c], *Vilém Neděla*[c], *Pavel Šácha*[d], *Martin Hrubý*[a]

[a] Institute of Macromolecular Chemistry, CAS

Heyrovského nám. 2, 162 06 Praha 6, Czech Republic

[b] Institute of Biochemistry and Experimental Oncology, First Faculty of Medicine

U Nemocnice 5, 128 53 Praha 2, Czech Republic

[c] Institute of Scientific Instruments, CAS

Královopolská 147, 612 00 Brno, Czech Republic

[d] Institute of Organic Chemistry and Biochemistry, CAS

Flemingovo nám. 2, 166 10 Praha 6, Czech Republic

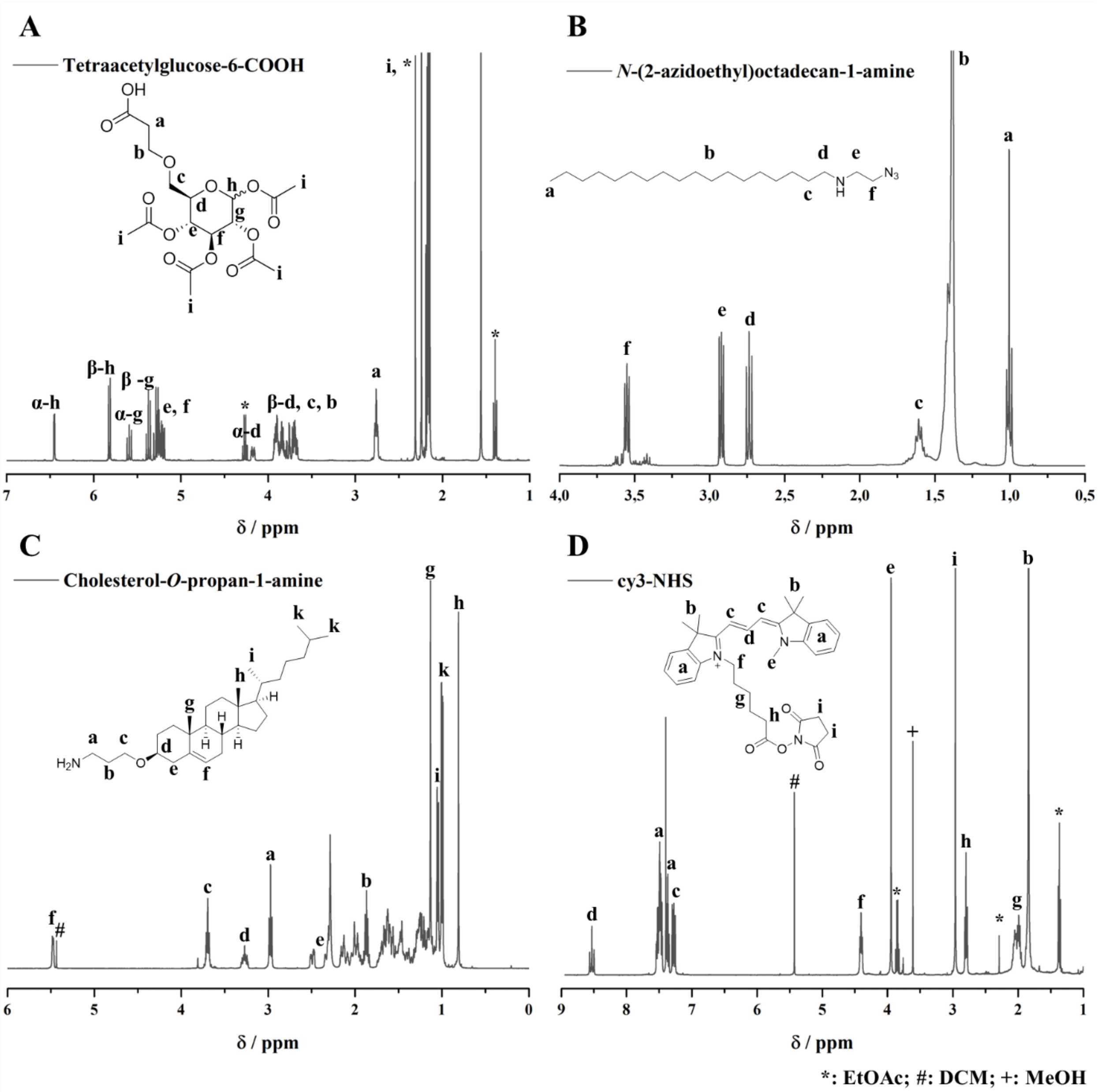

Figure S1. ^{1}H NMR spectra of tetraacetylglucose-6-COOH, used for the introduction of glucose side groups into the polyoxazoline polymers (A), *N*-(2-azidoethyl)octadecan-1-amine, used as a termination reagent in CROP of EtOx for P1 and P2 to introduce a hydrophobic C_{18} end group as well as a reactive azide group for the attachment of a fluorescent dye (B), cholesterol-O-propanamine, used to create a fluorescent cholesterol derivative (C), and cyanine 3 NHS ester for coupling with the latter (D). Both spectra were recorded in $CDCl_3$.

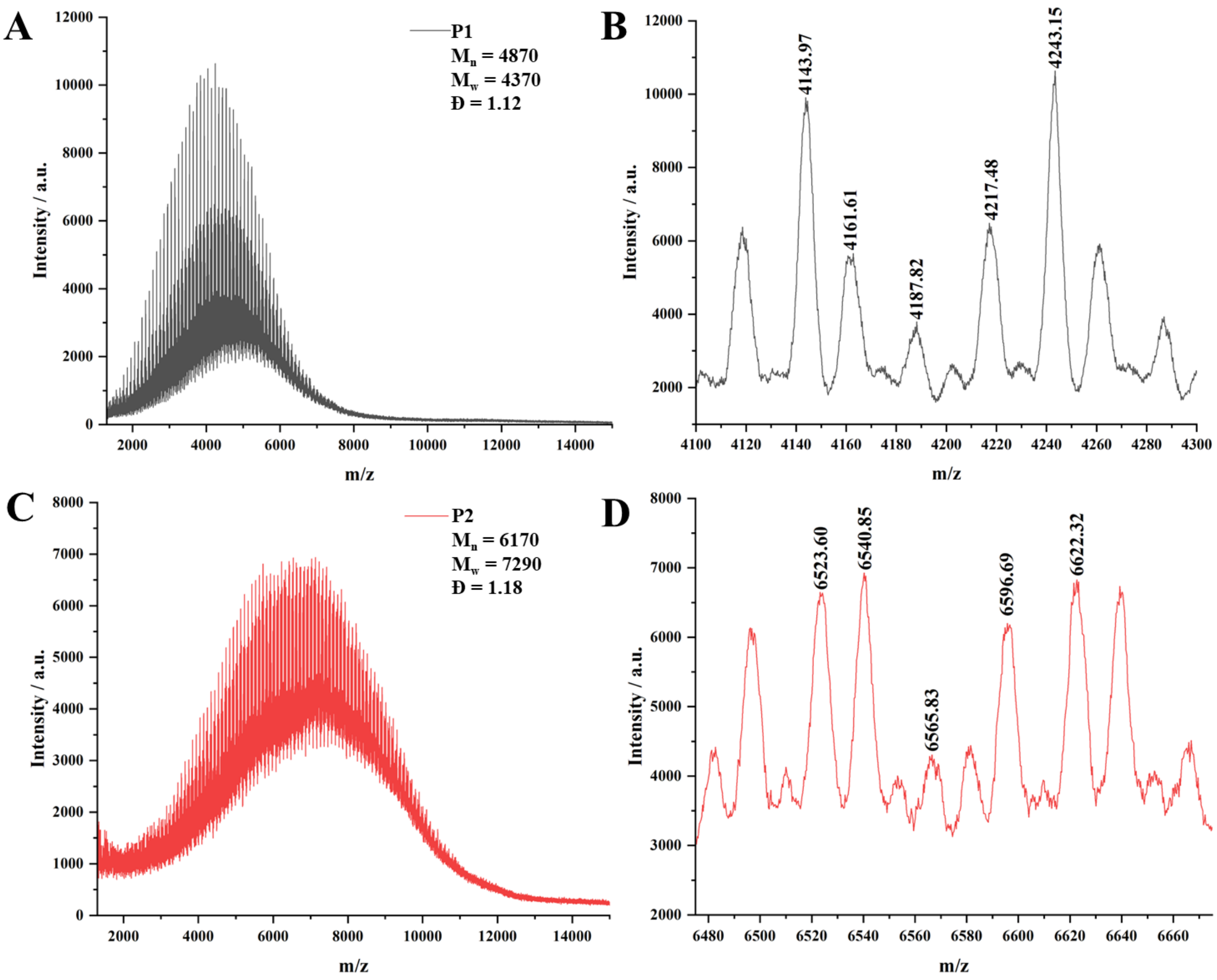

Figure S2. MALDI-TOF spectra including derived molar masses (M_n, M_w), dispersities (Đ), and magnified region for peak assignment of polymers P1 (A, B) and P2 (C, D). A description of the assigned peaks can be found in Table S1.

Table S1. Peak assignment of MALDI-TOF spectra shown in Figure S2.

Molecular weight in MALDI-TOF spectrum	Proposed structure and molecular weights
4243.15 g mol^{-1}; 6622.32 g mol^{-1}	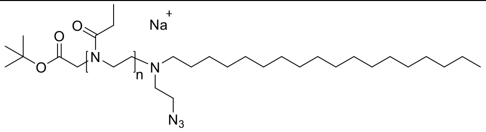 4242.8 g mol^{-1}; 6621.7 g mol^{-1}
4217.48 g mol^{-1}; 6596.69 g mol^{-1}	Azide fragmentation: 4216.8 g mol^{-1}; 6595.7 g mol^{-1}
4187.82 g mol^{-1}; 6565.83 g mol^{-1}	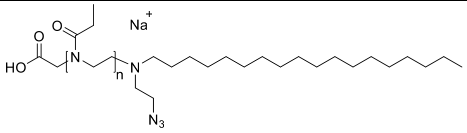 4186.7 g mol^{-1}; 6565.6 g mol^{-1}
4161.61 g mol^{-1}; 6540.85 g mol^{-1}	Azide fragmentation: 4160.7 g mol^{-1}; 6539.6 g mol^{-1}

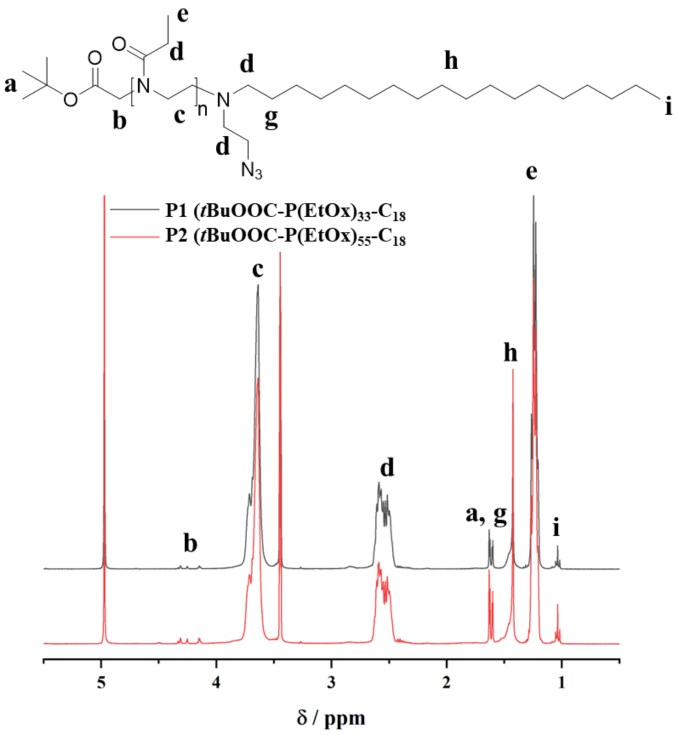

Figure S3. ^{1}H NMR spectra of polymers P1 and P2.

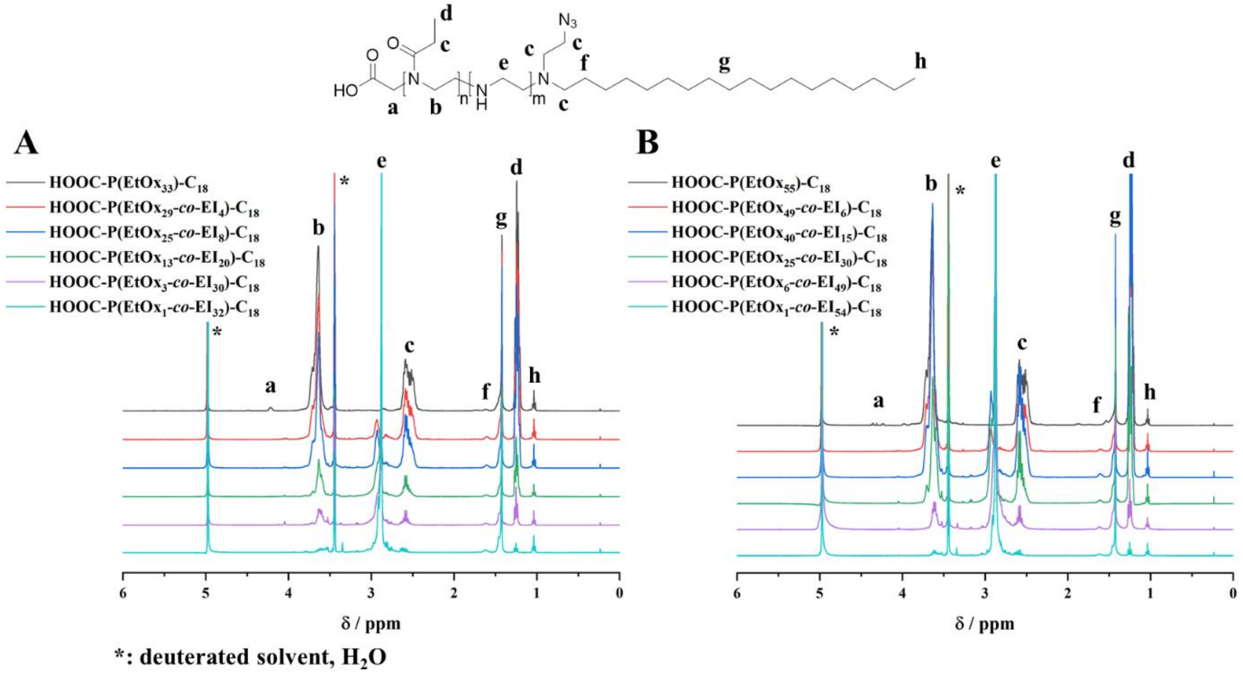

Figure S4. ^{1}H NMR spectra in MeOD of all polymers of the composition HOOC-P(EtOx$_{n\text{-}x}$-*co*-EI$_x$)$_n$-C$_{18}$, obtained *via* partial hydrolysis of polymers P1 and P2.

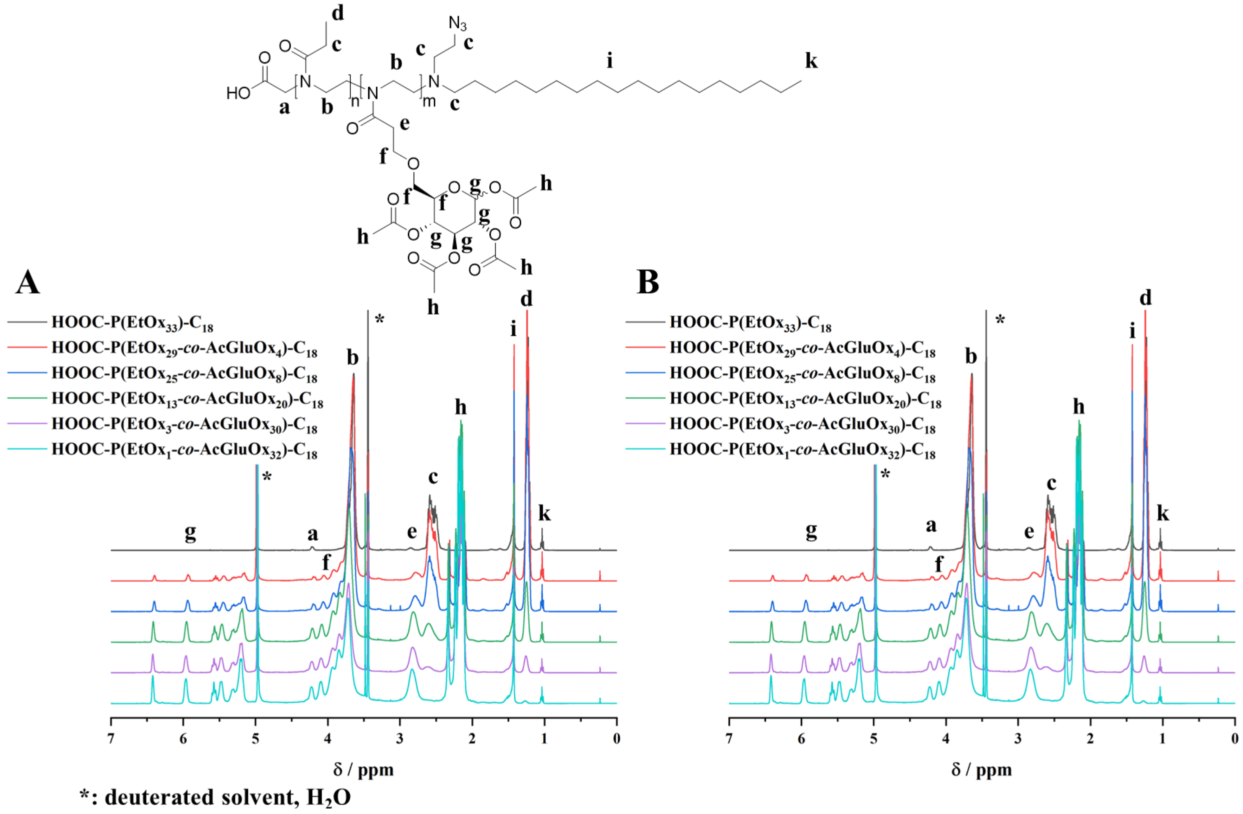

Figure S5. ^{1}H NMR spectra in MeOD of all polymers of the composition HOOC-P(EtOx$_{n\text{-}x}$-*co*-AcGluOx$_x$)$_n$-C$_{18}$, obtained *via* amide coupling reaction of HOOC-P(EtOx$_{n\text{-}x}$-*co*-EI$_x$)$_n$-C$_{18}$ with tetraacetylglucose-6-COOH.

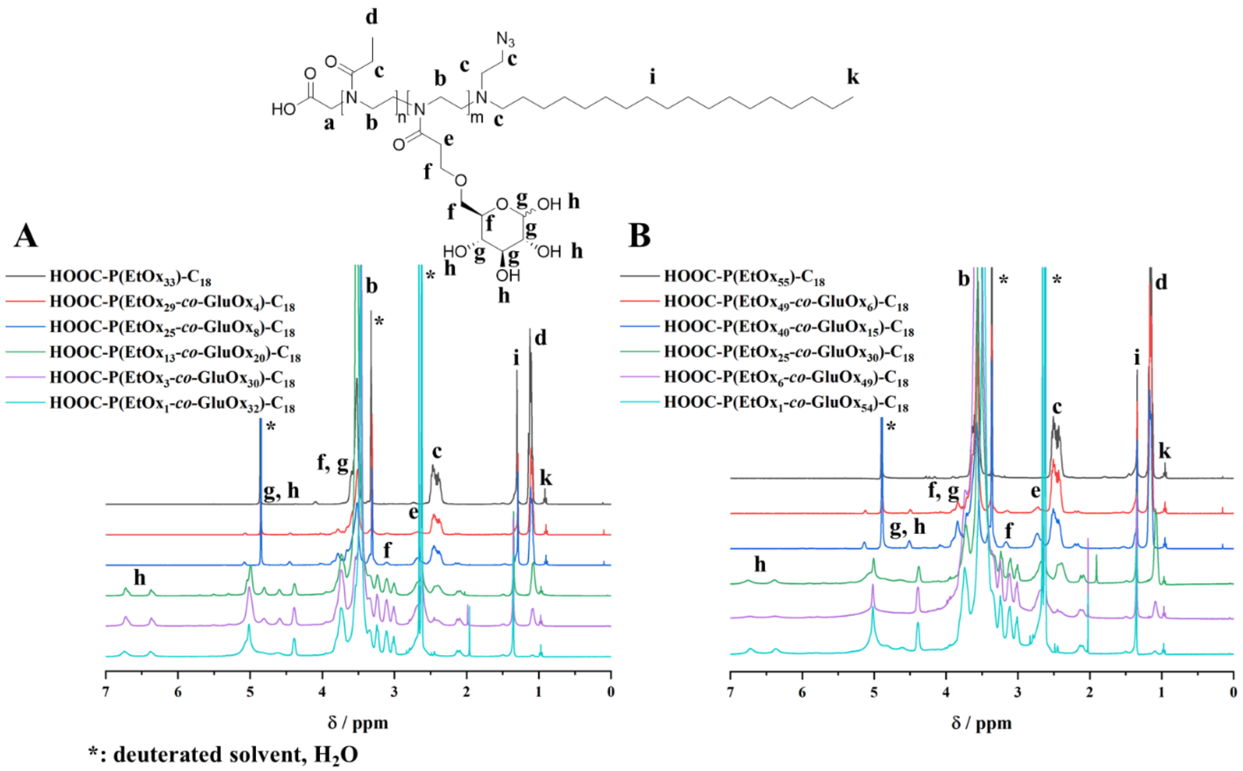

Figure S6. ^{1}H NMR spectra of all polymers of the composition HOOC-P(EtOx$_{n-x}$-*co*-GluOx$_x$)$_n$-C$_{18}$, obtained *via* basic deprotection of HOOC-P(EtOx$_{n-x}$-*co*-AcGluOx$_x$)$_n$-C$_{18}$. Due to differences in solubility of the end products, the spectra of P1-0 – P1-2 and P2-0 – P2-2 were recorded in MeOD, the spectra of P1-3 – P1-5 and P2-3 – P2-5 were recorded in DMSO-d$_6$.

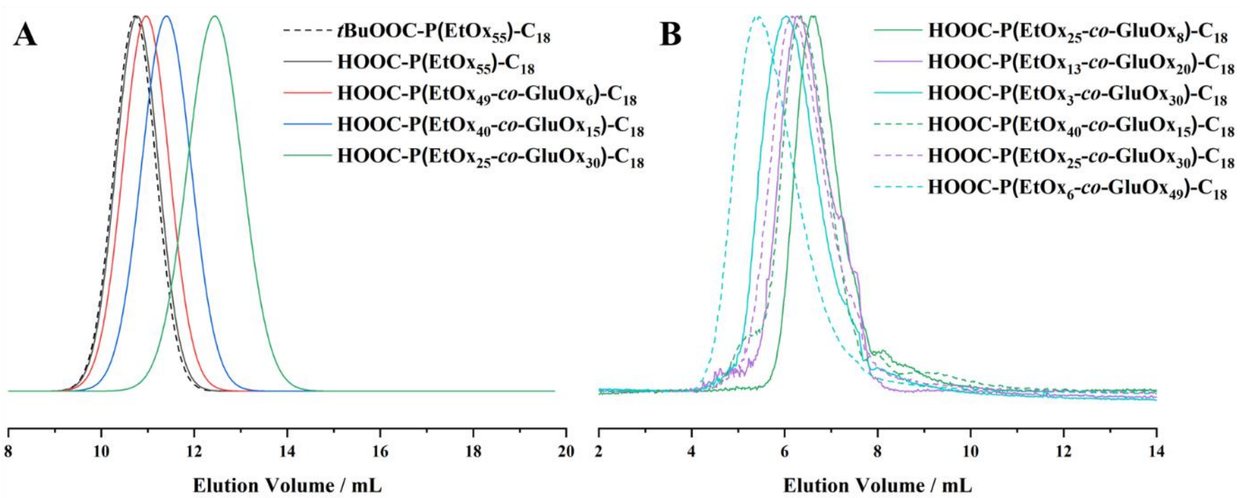

Figure S7. Refractive index (RI) GPC traces of P2-0 – P2-3 in MeOH/acetate buffer (pH = 6.0, 8:2) (A), and light scattering (LS) GPC traces of P1-3 – P1-5 and P2-3 – P2-5 in DMSO (B). The latter measurements were used for the determination of molecular weights and dispersities listed in table 2.

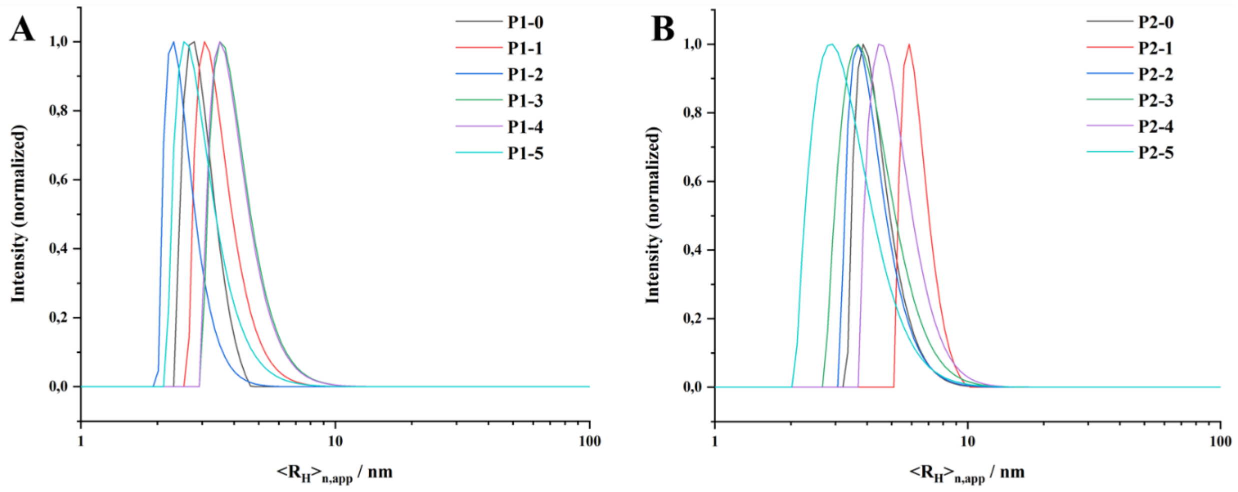

Figure S8. Number-weighted DLS CONTIN plots of P1-0 – P1-5 and P2-0 – P2-5 in aqueous solution (1 mg mL^{-1}). The low hydrodynamic radii indicate no formation of defined micelles.

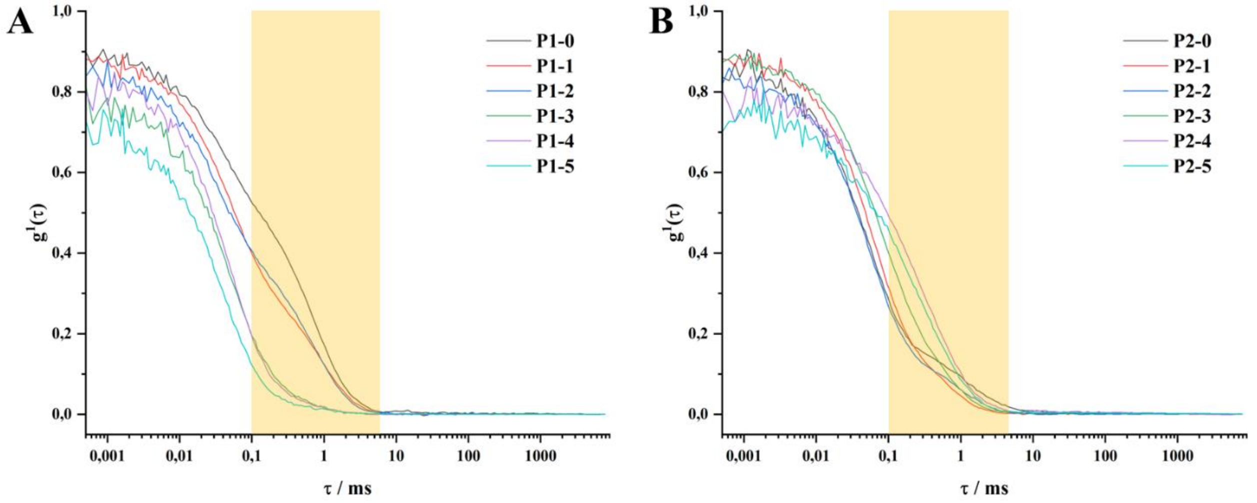

Figure S9. Correlation functions of DLS measurements of the polymers in aqueous solutions at 1 mg mL^{-1}. Despite the fact that the number-weighted CONTIN plots show no significant aggrgation, the presence of the shoulder in the correlation function (yellow area, especially for compounds with a lower degree of functionalization) indicates the presence of another species of particles with a different size, probably loose aggregates.

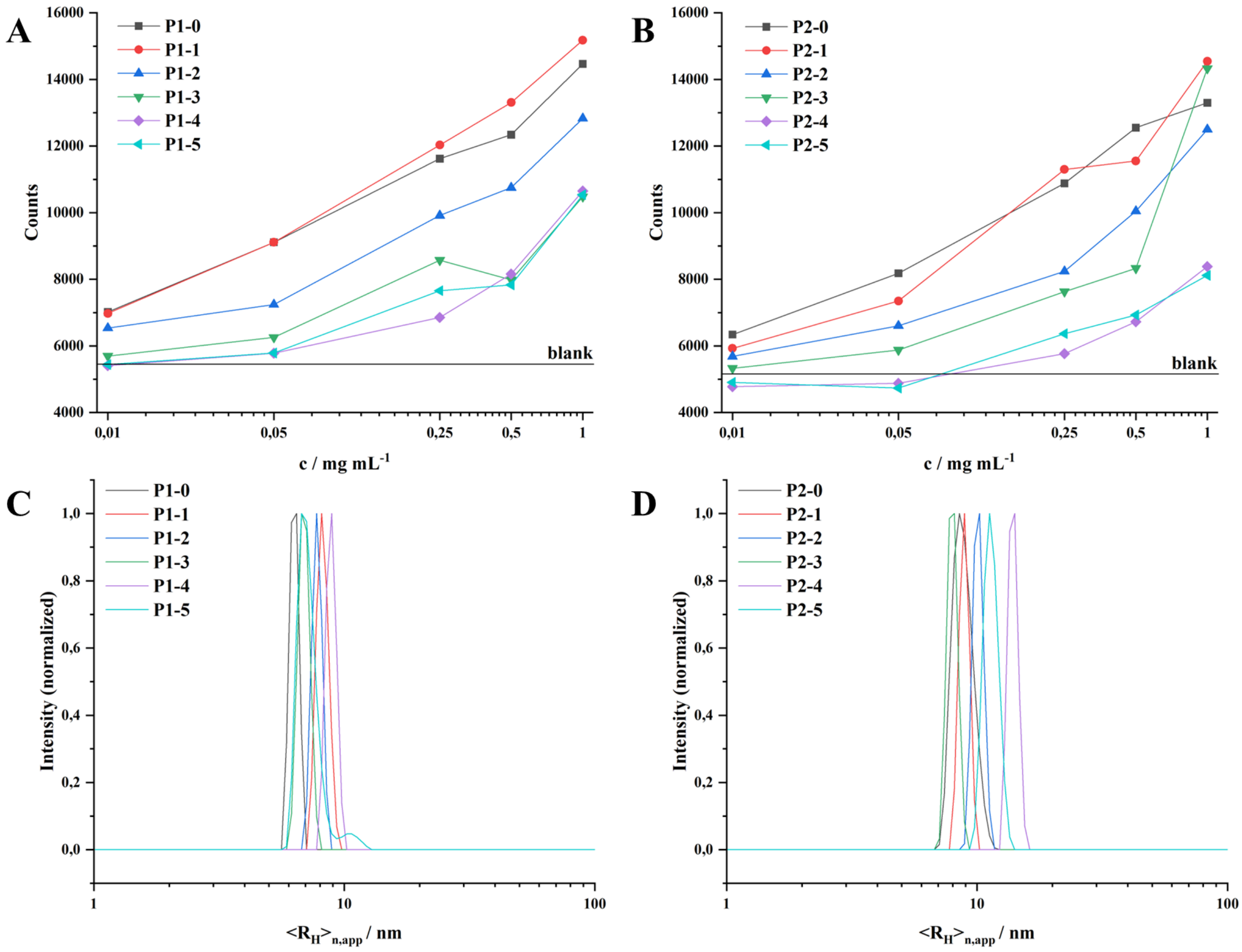

Figure S10. Investigation of the micellization behavior of the polymers in DMEM at 37 °C. All polymers were able to encapsulate nile red, which is visible due to the increased fluorescence of polymer-nile red solutions in comparison to pristine nile red solutions (A, B). This proves the formation of micelles in the chosen conditions. For P1-3 – P1-5 and P2-3 – P2-5, a sudden change in rate of the increase of the fluorescence intensity suggests that the critical micelle concentration was reached in between 0.25 and 0.5 mg mL^{-1}. All polymers do form micelles at 1 mg mL^{-1} at 37 °C in DMEM, which is demonstrated in DLS measurements. The number-weighted CONTIN plots are shown (C, D).

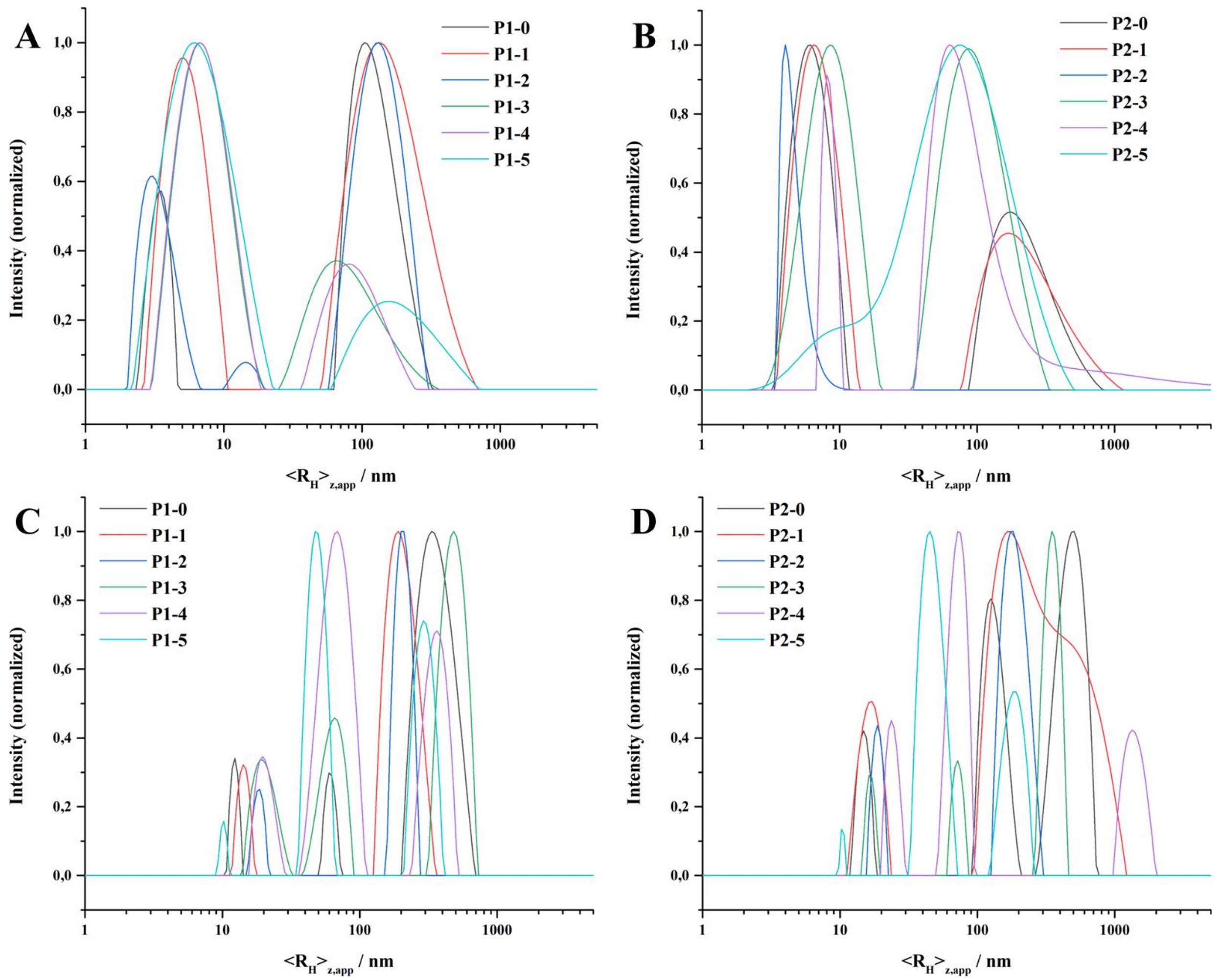

Figure S11. Intensity-weighted DLS CONTIN plots of pure polymers in water (1 mg mL^{-1}, A and B), and SLNP in water (1 mg mL^{-1} polymer with 0.4 mg mL^{-1} lipid, C and D).

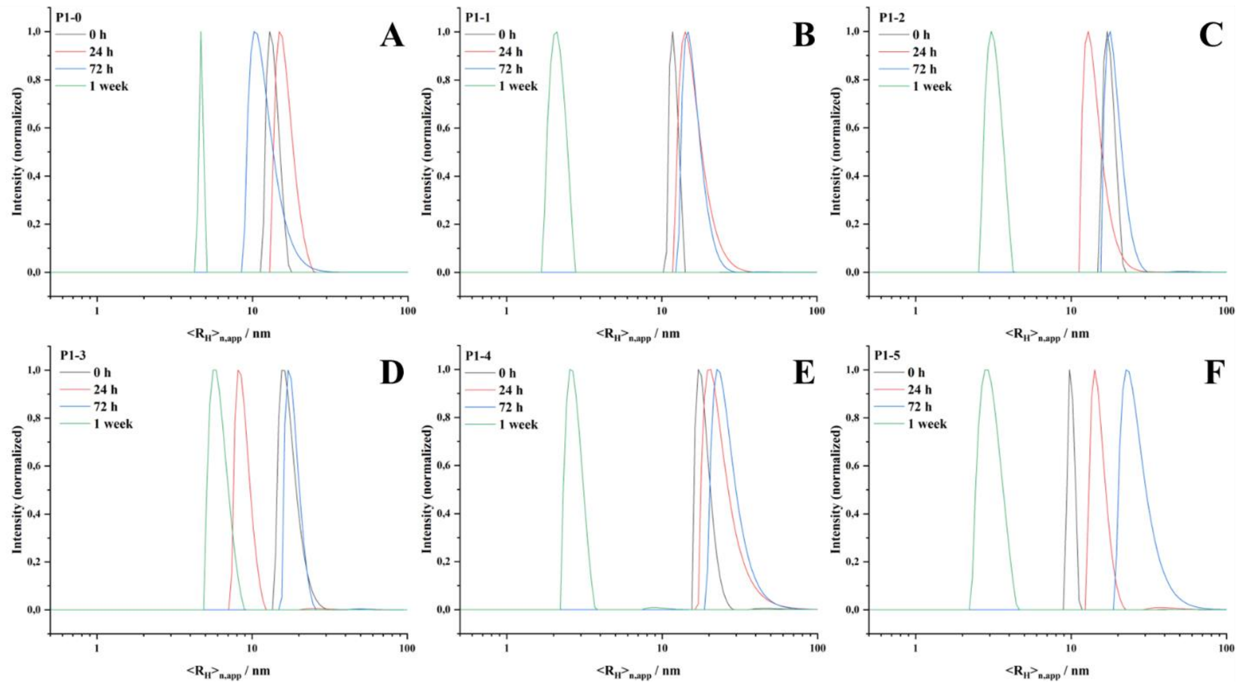

Figure S12. Number-weighted DLS CONTIN plots of SLNP prepared from P1-0 – P1-5 at different time points after preparation. The majority of the particles decomposed after 1 week, which is indicated by a shift of $\langle R_H \rangle_{n,app}$ to lower values.

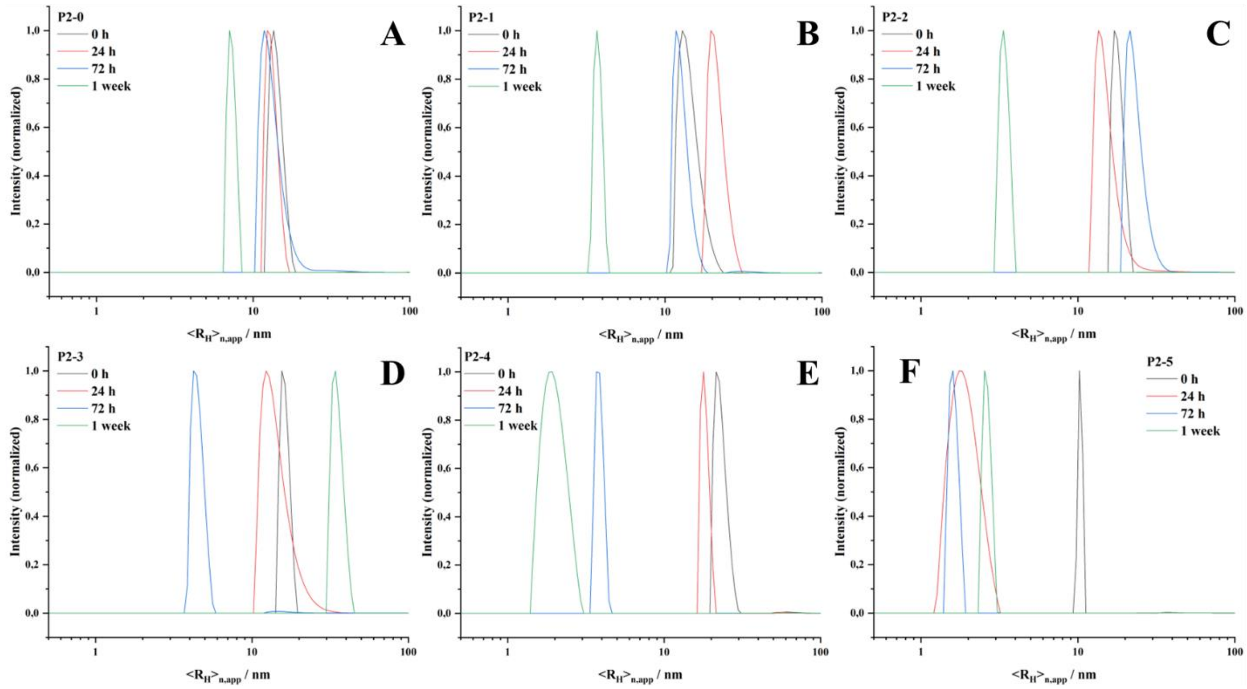

Figure S13. Number-weighted DLS CONTIN plots of SLNP prepared from P2-0 – P2-5 at different time points after preparation. The majority of the particles decomposed after 1 week, which is indicated by a shift of $\langle R_H \rangle_{n,app}$ to lower values. Faster decomposition is observable for SLNP prepared from P2-4 and P2-5 (E, F). For P2-3 (D), the $\langle R_H \rangle_{n,app}$ of the measurement after 1 week was shifted to larger hydrodynamic radii due to incomplete sedimentation.

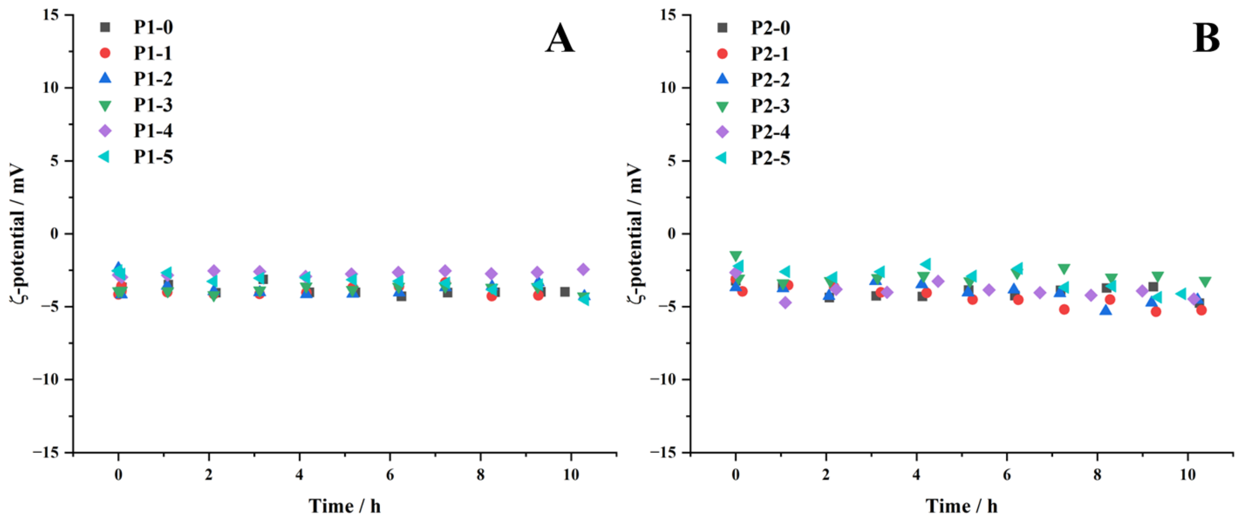

Figure S14. Zeta potential in mV of SLNP in DMEM over time.

Table S2. Stability of the SLNP in DMEM as a preliminary test to ensure the integrity of the particles during biological experiments. The times in this table were determined using the change in the increase of the unweighted hydrodynamic radius (see Figure S14).

Polymer	Stability / h	Polymer	Stability / h
P1-0	2	P2-0	12
P1-1	7	P2-1	12
P1-2	12	P2-2	3
P1-3	12	P2-3	1
P1-4	9	P2-4	7
P1-5	12	P2-5	12

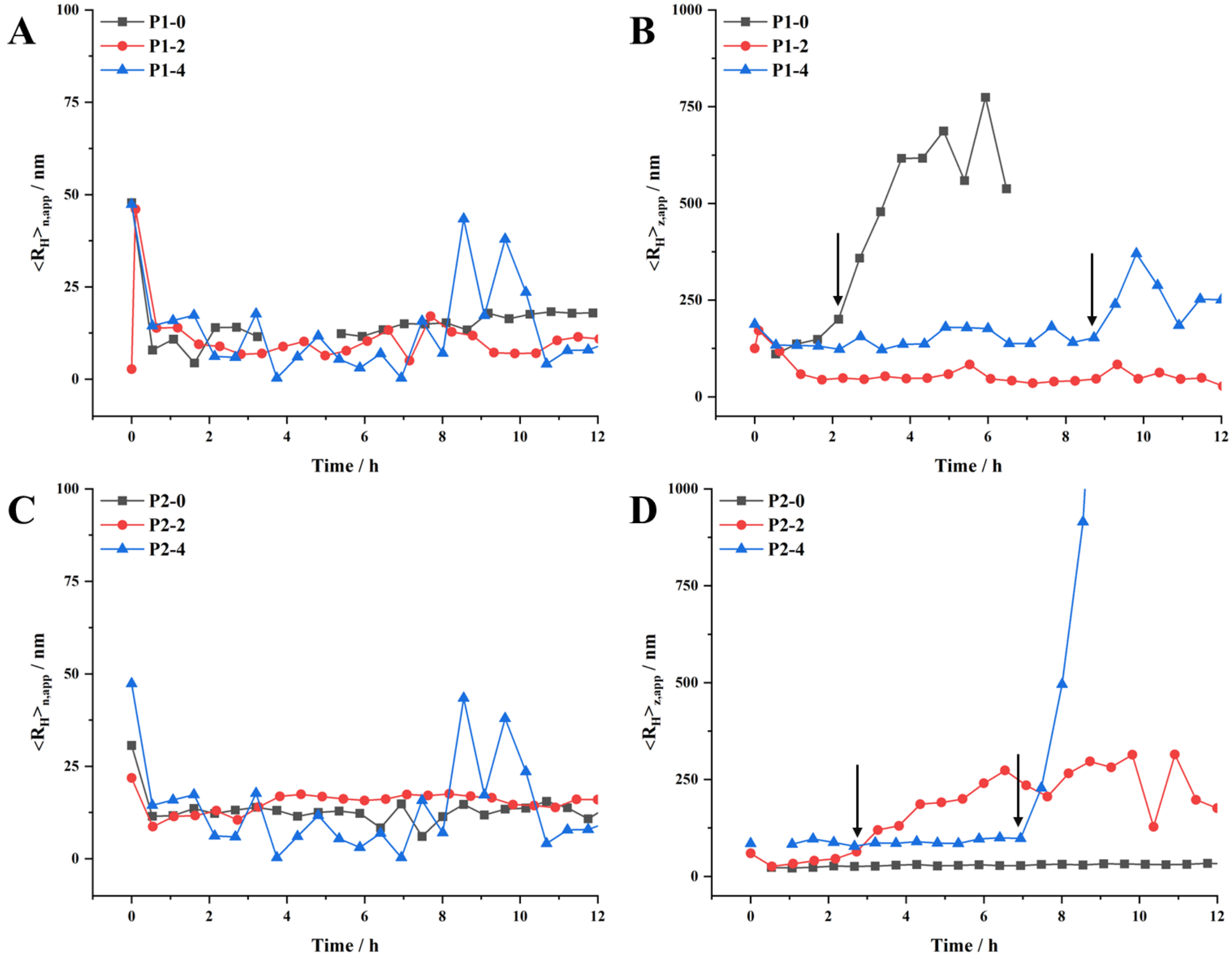

Figure S15. Number- and intensity-weighted hydrodynamic radii of selected SLNP at different time points after preparation and subsequent dilution of the samples with DMEM (9:1). The black arrows indicate time points after which a a significant increase in hydrodynamic radius indicates the aggregation of SLNP due to the displacement of the stabilizer. This will lead to the precipitation and sedimentation of the lipid over time, leaving behind the dissolved stabilizer as micelles in DMEM solution.

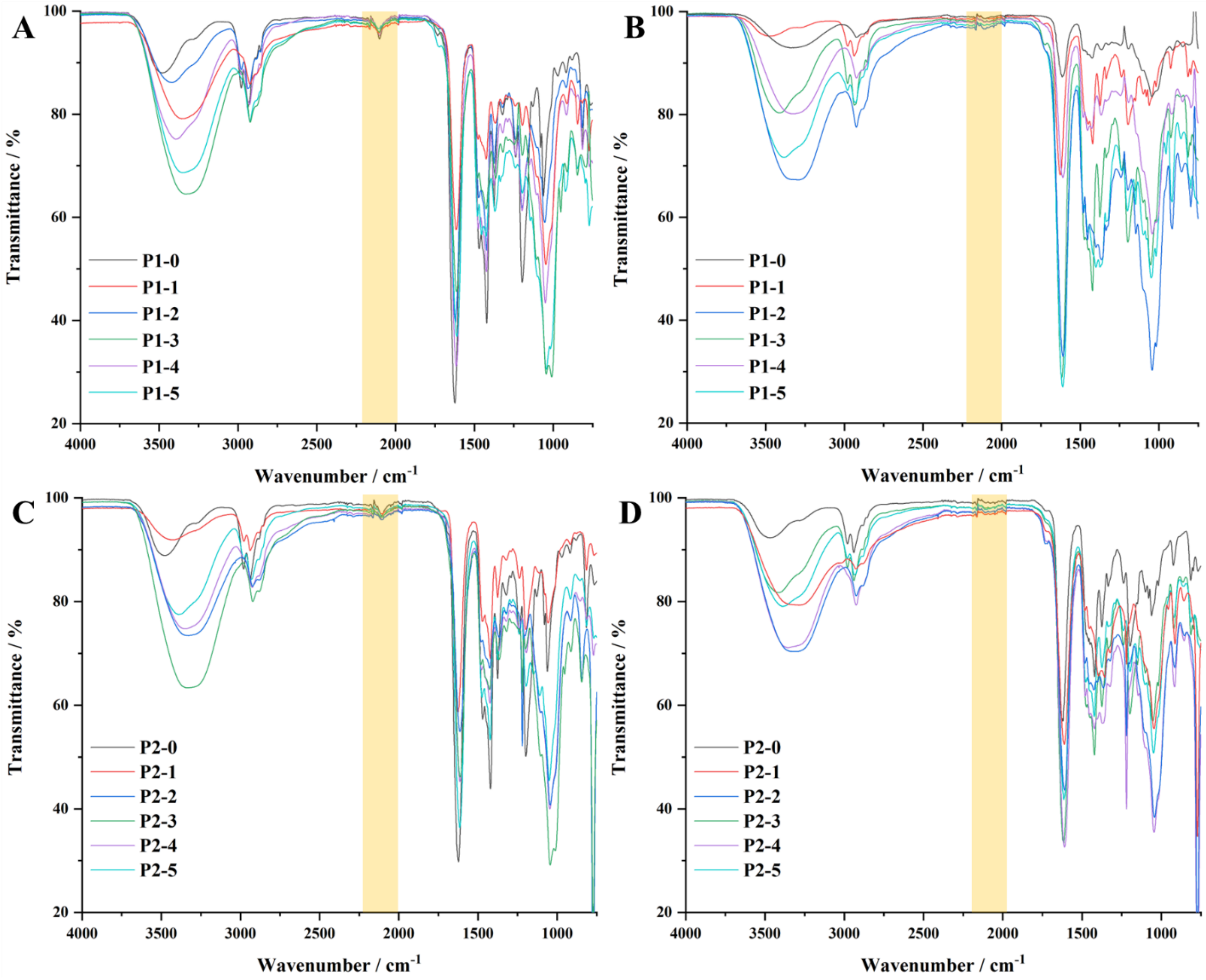

Figure S16. FTIR spectra of P1 and P2 before (A, C) and after (B, D) CuAAC with the alkyne-functionalized cyanine 5 dye. The area of the characteristic azide stretching vibration is marked in yellow.

Table S3. Degree of functionalization of all glycopolymers with cy5, as determined by UV/vis spectroscopy in micropure water using λ_{max} = 642 nm. The extinction coefficient of the reactive dye was determined experimentally to be ε = 85609 cm^{-1} mol^{-1}.

Polymer	DoF / %	Polymer	DoF / %
P1-0	25	P2-0	22
P1-1	22	P2-1	18
P1-2	40	P2-2	27
P1-3	55	P2-3	45
P1-4	59	P2-4	58
P1-5	34	P2-5	36

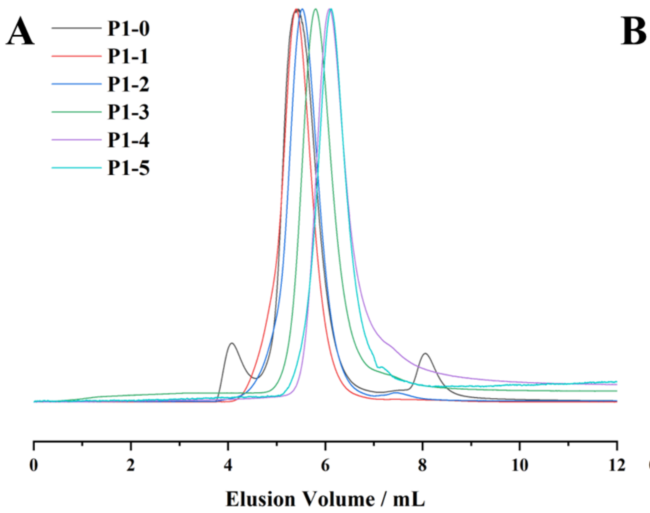

Figure S17. UV GPC traces of all glycopolymers that show the absence of free cy5 dyes in all samples except for P1-0, which contained a small amount of free dye.

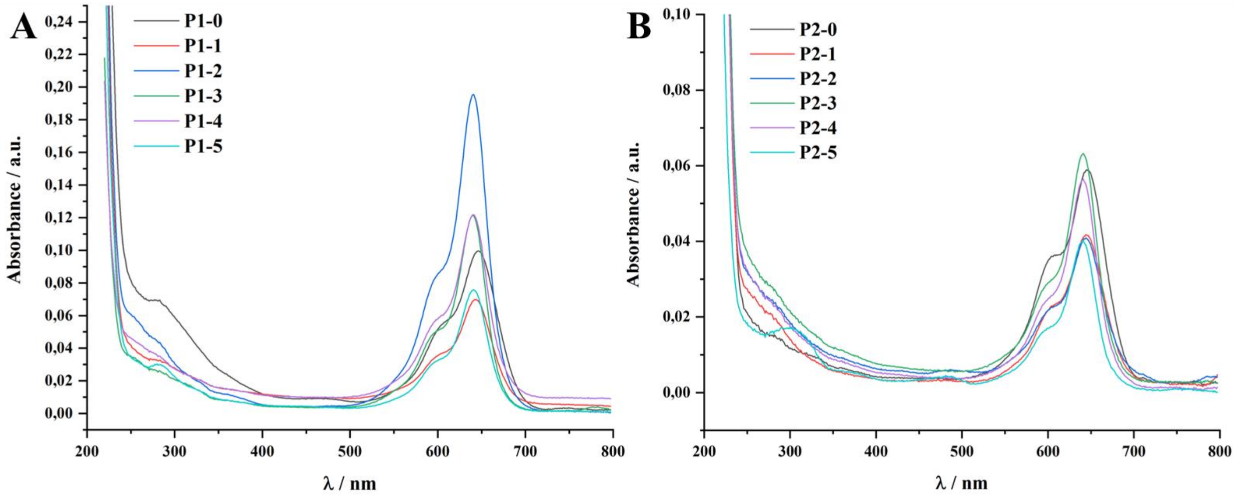

Figure S18. UV/vis spectra of glycopolymers P1-0 – P1-5 (B) and P2-0 – P2-5 (C) in aqueous solution.

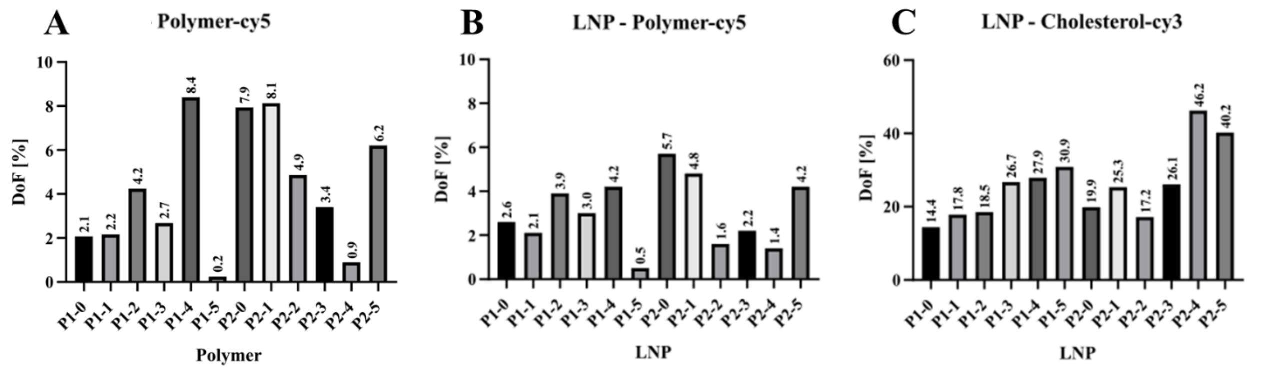

Figure S19. Degree of functionalization (DoF) of polymers with cy5 (A) and SLP with cy5 (polymer-cy5, B) and cy3 (cholesterol-cy3, C) as determined via UV/vis spectrometry prior to biological studies with the respective samples.

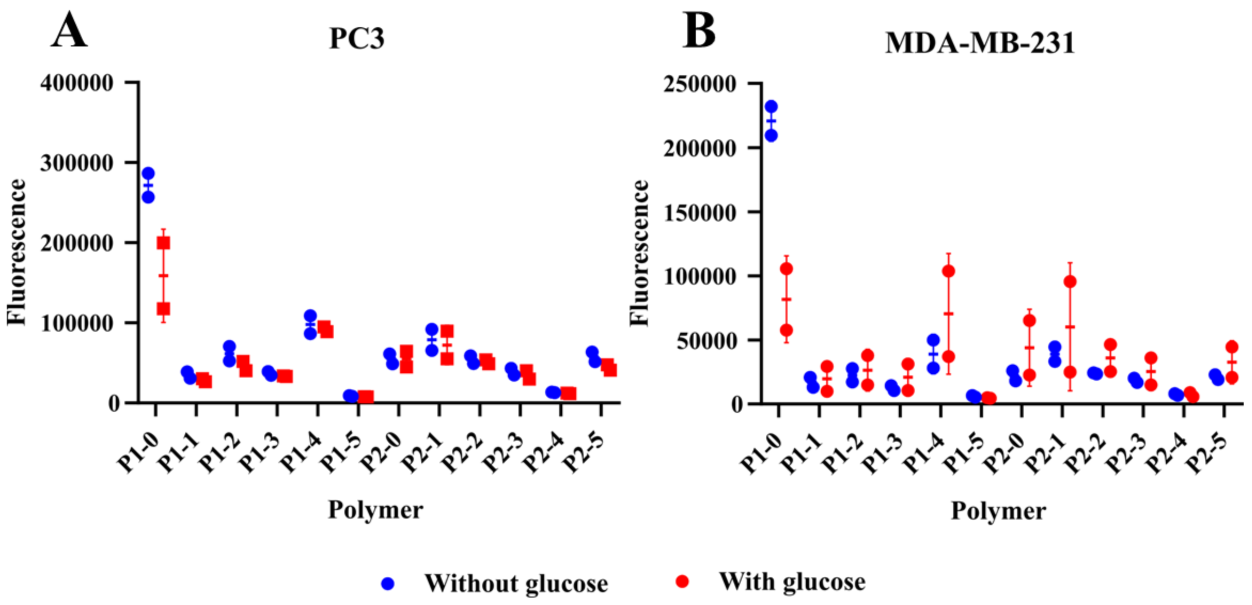

Figure S20. Non-normalized fluorescence in flow cytometry experiments with pristine polymers and PC3 (A) or MDA-MB-231 cells (B) for the investigation of polymer-cell interactions.

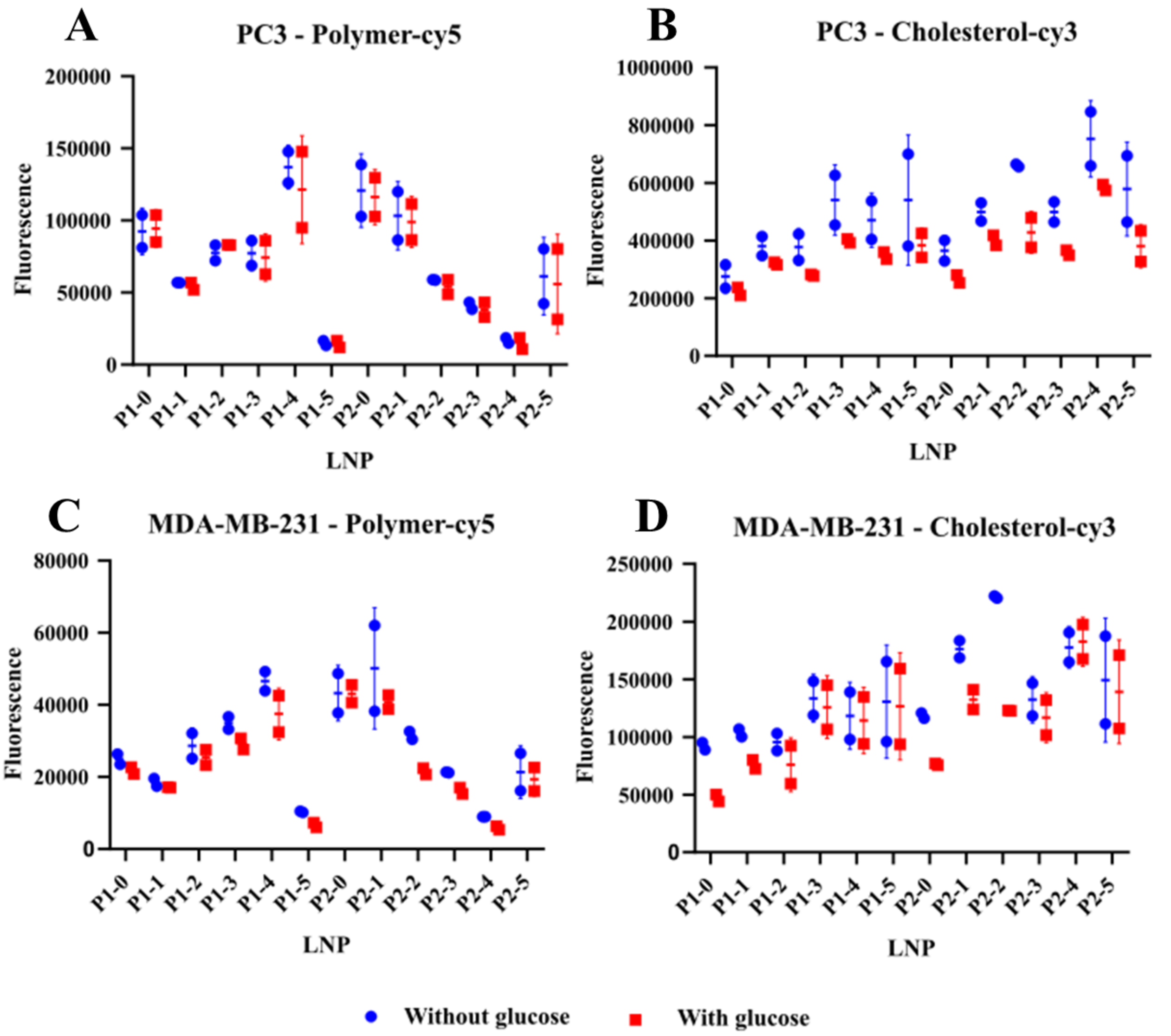

Figure S21. Non-normalized fluorescence in flow cytometry experiments with LNP and PC3 (A, B) or MDA-MB-231 cells (C, D). The fluorescence was measured seperately for cy5 (polymer-cy5, A, C) and cy3 (cholesterol-cy3, B, D).
